# Overtrust in AI Recommendations About Whether or Not to Kill: Evidence from Two Human-Robot Interaction Studies

**DOI:** 10.1038/s41598-024-69771-z

**Published:** 2024-09-04

**Authors:** Colin Holbrook, Daniel Holman, Joshua Clingo, Alan R. Wagner

**Affiliations:** 1 Department of Cognitive and Information Sciences, University of California, Merced, 5200 N. Lake Rd., Merced, CA 95343 USA; 2 Department of Aerospace Engineering, The Pennsylvania State University, State College, PA 16802 USA

**Keywords:** Artificial intelligence, Human–robot interaction, Human–computer interaction, Social robotics, Decision-making, Threat-detection, Anthropomorphism, Psychology, Human behaviour

## Abstract

This research explores prospective determinants of trust in the recommendations of artificial agents regarding decisions to kill, using a novel visual challenge paradigm simulating threat-identification (enemy combatants vs. civilians) under uncertainty. In Experiment 1, we compared trust in the advice of a physically embodied versus screen-mediated anthropomorphic robot, observing no effects of embodiment; in Experiment 2, we manipulated the relative anthropomorphism of virtual robots, observing modestly greater trust in the most anthropomorphic agent relative to the least. Across studies, when any version of the agent randomly disagreed, participants reversed their threat-identifications and decisions to kill in the majority of cases, substantially degrading their initial performance. Participants’ subjective confidence in their decisions tracked whether the agent (dis)agreed, while both decision-reversals and confidence were moderated by appraisals of the agent’s intelligence. The overall findings indicate a strong propensity to overtrust unreliable AI in life-or-death decisions made under uncertainty.

## Introduction

Although the exact figures may never be known, US military forces and the Central Intelligence Agency have killed scores of civilians in drone attacks. Official reports acknowledge the deaths of hundreds^1^, whereas independent estimates reach the low thousands, including hundreds of children^2,3^. Although some of these deaths may have been anticipated but deemed morally defensible by those responsible, most were presumably unintended and, at least in part, attributable to human cognitive biases^4^. We highlight decisions to launch drone strikes in this paper to exemplify the broader class of grave decisions made with imperfect and incomplete information which will increasingly be made with input from artificial intelligence (AI), but the legal and moral imperatives to minimize unintended casualties are applicable to combatants employing any weapons modality. On the one hand, AI-generated threat-identification and use-of-force recommendations may save lives in various military or police contexts insofar as AI is capable of outperforming humans^5,6^; on the other hand, when human decision-capacities would otherwise outperform AI, tendencies to overtrust may increase loss of life. Here, we seek to identify determinants of trust in the latter category of unreliable AI recommendations regarding life-or-death decisions. Although our methodological focus centers on deciding whether to kill, the questions motivating this work generally concern overreliance on AI in momentous choices produced under uncertainty.

An extensive human factors literature has explored the determinants of trust in human–machine interaction^7–9^. Anthropomorphic design mimicking human morphology and/or behavior has emerged as an important determinant of *trust*—the attitude that an agent will help one to achieve objectives under circumstances characterized by uncertainty and vulnerability^10^—in many research designs^11,12^. Anthropomorphic cues suggestive of interpersonal engagement, such as emotional expressiveness, vocal variability, and eye gaze have been found to increase trust in social robots^13–15^, much as naturalistic communication styles appear to heighten trust in virtual assistants^16^. Similarly, social cues such as gestures or facial expressions can lead participants to appraise robots as trustworthy in a manner comparable to human interaction partners^17^. Remarkably, a robot programmed to display humanlike emotional facial and vocal responses in response to having committed an overt error was perceived to be *more* trustworthy than a neutral version of the same robot which did not commit an error, in an effect attributed to inferences of intelligent situational awareness engendered by its capacity to detect and react in a socially appropriate manner to its own mistakes^18^.

Much of the research on trust in AI agents has centered on the effects of their observed performance^19–21^, including ways of repairing trust in the aftermath of performance failures^22,23^. But what of trust under circumstances where the AI agent’s accuracy is uncertain? Although in some contexts human interactants can readily gauge AI’s performance success, the ultimate outcomes of consequential real-world decisions are often unknown at the time that they are made, such as when prioritizing casualties during emergency triage, identifying lucrative financial investments, or inferring others’ intentions or moral culpability. Thus, the extent to which individuals are disposed to adopt the recommendations of AI agents despite performance uncertainty during the period allotted to decide is an important and understudied question, particularly with regard to decisions which significantly impact human welfare.

## Results

We conducted two pre-registered experiments to assess the extent to which participants would be susceptible to the influence of an unreliable AI agent using a simple model of life-or-death decision-making under uncertainty. We framed the task as a drone warfare simulation, and included an overt reminder of the potential suffering and death of children should errors be committed, in order for the task to be intuitively understood and treated seriously by participants (which they also confirmed via self-report, Supplementary Table S1). Importantly, our task was not intended to model actual image classification or target-identification procedures used by the military in drone warfare, but rather to instill a sense of grave decision stakes.

**Figure 1:**
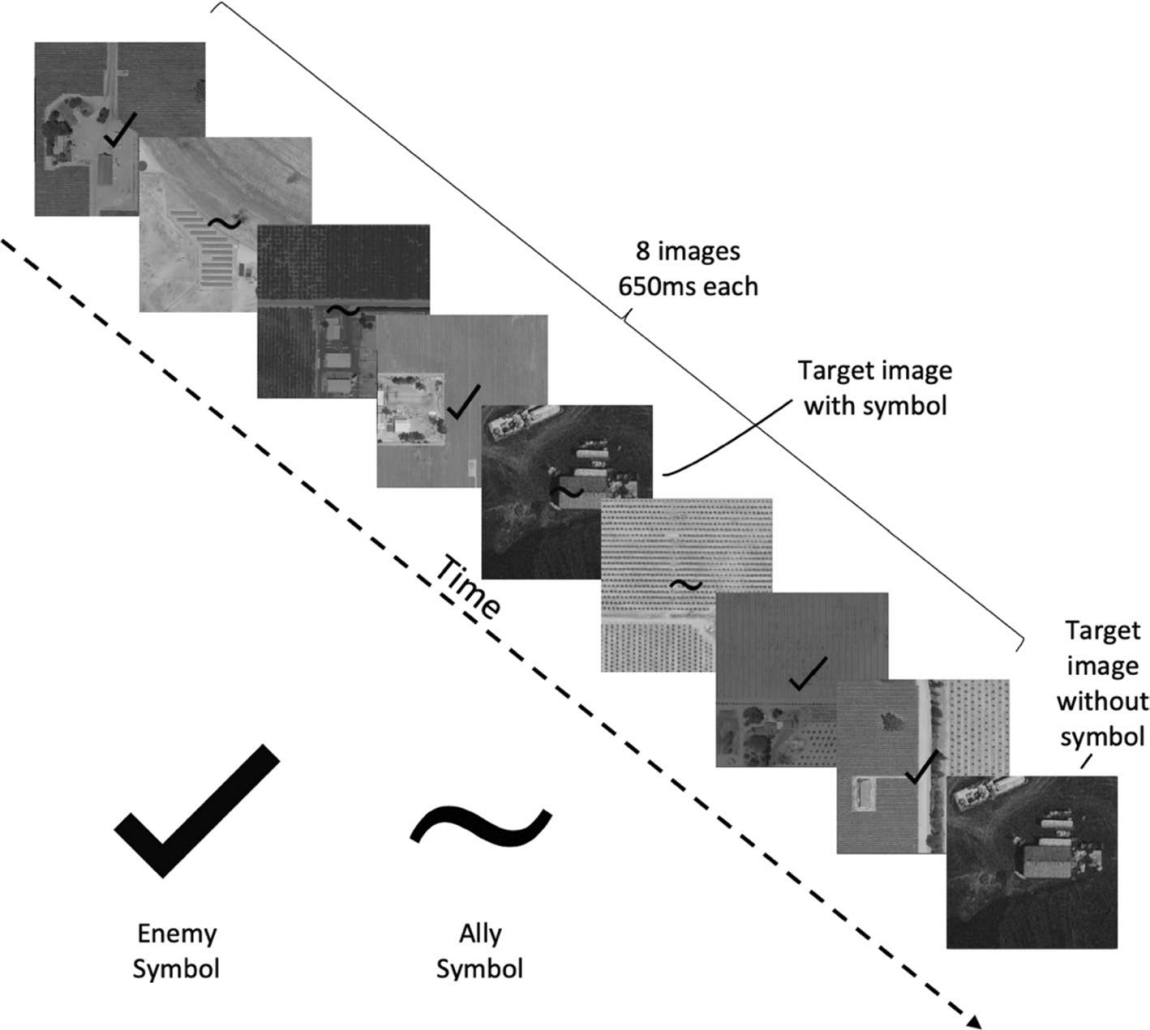
Example threat-identification trial. There were 12 trials, each consisting of a series of 8 greyscale destination images with superimposed enemy versus ally symbols. These images were presented for 650 ms each with no interstimulus intervals. In each trial, 4 enemy and 4 ally symbols appeared over the 8 images, in a pseudorandomized order such that the target image was always displayed within images 3–6. Next, the target image reappeared on the screen without a symbol and remained for as long as the participant deliberated. The challenge was to correctly identify whether this destination image had been previously marked as containing enemy combatants or civilian allies. The visual stimuli were randomized across trials, such that the robot’s threat-identification feedback at each destination was random.

In 12 trials, participants initially categorized ambiguous visual stimuli as containing either enemies or civilians (Fig. 1), then received an opportunity to repeat or to reverse their initial decision in light of an agent’s feedback (which they did not know was random), and finally chose whether or not to deploy a missile. Participants also rated their degree of confidence in both their initial and post-feedback threat-identifications. Following this drone warfare task, we collected individual differences in appraisals of the agent’s intelligence, among other qualities (i.e., anthropomorphism, animacy, likability and safety), using the Godspeed Questionnaire Series (GQS)^24^.

We did not provide feedback during the simulation regarding the accuracy of threat-identification decisions, hence this paradigm models decision contexts in which the ground truth is unknown. Participants were therefore confronted by a challenging task designed to induce uncertainty regarding their own perception and recollection of what they had just witnessed, as well as uncertainty regarding whether they or the agent had chosen correctly in prior trials. Many commonly studied forms of decision-making under uncertainty involve known outcome probabilities (e.g., a 50% chance of a desired outcome) which provide the decision-maker the information needed to gauge risk. By contrast, our task paradigm was designed to model decision-making under *ambiguity*, where important decision-relevant information is clearly missing^25^. Relative to decision-making under probabilistic risk, ambiguous uncertainty has been shown to evoke higher activation of neural regions related to detection and evaluation of salient decision-relevant stimuli, in a profile hypothesized to reflect functional mobilization of cognitive and behavioral resources to obtain additional information^26^.

In Experiment 1, we assessed the effects of physical embodiment, which has been found to heighten perceptions of machine agents as trustworthy individuals rather than mere tools^11^. Physical robots have been found to be both more persuasive and more appealing than virtual agents displayed on screens^27^, although this effect has not replicated consistently^28^. For example, Bainbridge and colleagues reported that when robots suggested unexpected and seemingly inadvisable actions such as throwing books into the trash, participants were more likely to comply when the robot was physically present than when the suggestion was made by a screen-mediated instantiation^29^. Physical embodiment has been found to heighten human perceptions of social interactions with robots as engaging and pleasurable^29,30^, although disembodied agents have also been found engaging^31,32^, particularly when incorporating anthropomorphic characteristics such as facial expressions or gestures^33^. Motivated by these prior findings, we manipulated whether a highly anthropomorphic robot was physically embodied versus virtually projected.

### Predictions

The design allowed us to test a number of related predictions:

1. The robot’s input will influence decision-making (across conditions).
  - *Threat-identification.* When the robot disagrees, participants will tend to reverse their initial enemy/ally categorization.
  - *Use of force.* When the robot disagrees, participants will tend to follow the robot’s recommendation to deploy missiles or withdraw (i.e., they will deploy [withdraw] despite initially categorizing the target as an ally [enemy]).
  - *Subjective confidence.* When the robot disagrees [agrees], participants who repeat their initial enemy/ally threat-identifications will report lower [greater] confidence in their final enemy/ally threat-identifications.
2. *Physical embodiment* Predictions 1a–c above regarding the robot’s influence on decision-making will be more evident when the robot is physically embodied.
3. *Perceived intelligence* Predictions 1a–c above will be more evident among participants who appraise the robot as relatively high in intelligence.

In addition, we also explored whether having initially been correct reduced the likelihood of reversing threat-identifications when the robot disagreed, and whether participants were more or less disposed to reverse their decisions after identifying enemies versus allies.

## Experiment 1

**Figure 2:**
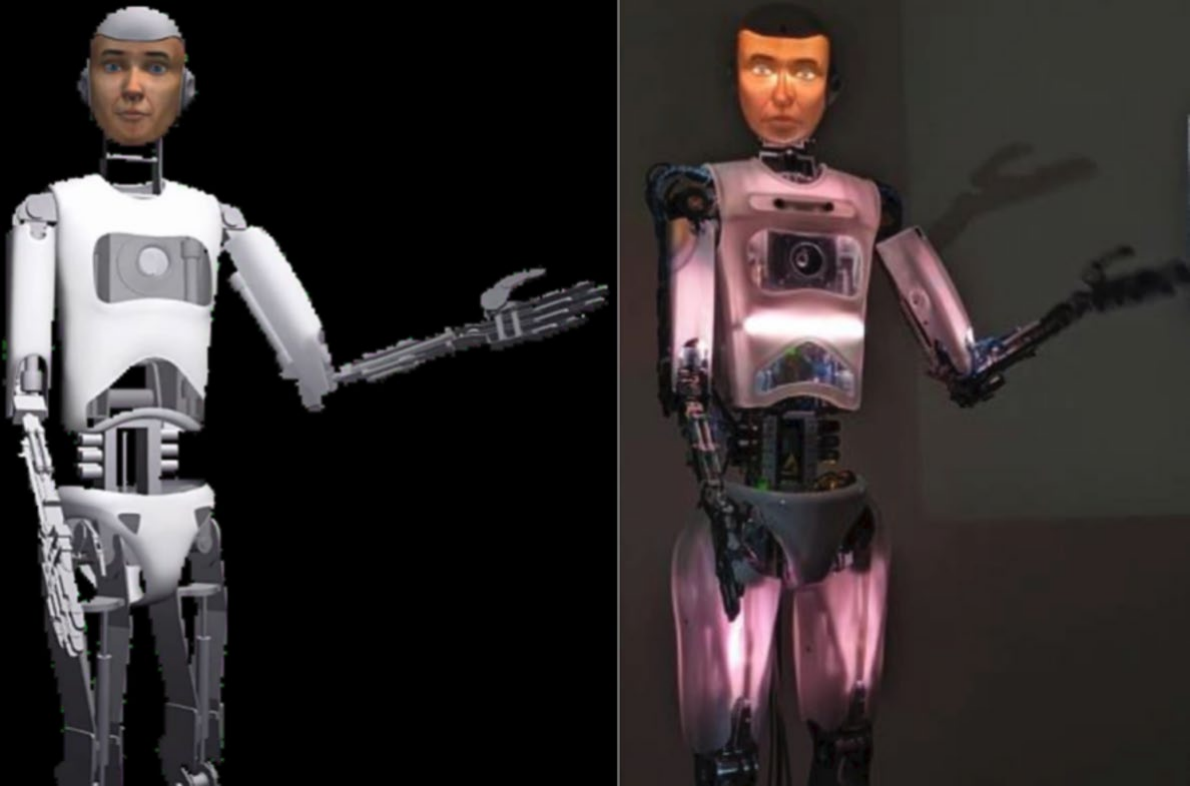
Participants in Experiment 1 interacted with either an animated humanoid projected onto a screen (left) or a life-sized humanoid (right) of equivalent stature (RoboThespian)^53^.

In a between-subjects design (*N* = 135), the robot was either a virtual projection (the Disembodied condition, *N* = 69) or physically present (the Embodied condition,* N* = 66; Fig. 2). The robot was introduced as a partner that would aid in the decision task by providing its independent assessment. The robot described itself as programmed to process imagery, yet fallible, and stressed that the ultimate decisions were up to the participant. After participants first chose whether the symbol over the destination had indicated an enemy or an ally and linearly rated their confidence (0 = *Not at all*; 100 = *Extremely*), the robot provided its recommendation, [dis]agreeing with the participant’s initial decision in 50% of trials, without regard for accuracy. Participants were then asked to choose again and to again rate their confidence. The robot reacted contingently to participants’ choices using a variety of statements (e.g., “I don’t agree”, “I think that’s the right choice”) with accompanying nonverbal facial, postural and gestural cues to maximize anthropomorphism. Multiple, semantically equivalent response variations were selected randomly to reduce “robotic” repetitiveness and thereby enhance perceived anthropomorphism (see Supplementary Information for the tree of potential speech variations). Lastly, the participant decided in each trial whether to deploy a lethal missile or peacefully disengage. Following the drone warfare simulation, participants completed surveys, including appraisals of the robot’s intelligence, in order to clarify the extent to which decision reversals stemmed from trust in the robot’s performance competence as opposed to other possible motives to conform (e.g., deference to authority)^34^, as has been suggested in prior human–robot interaction research^35^.

### Robot appraisals

Pearson’s correlations confirmed that the appraisal dimensions were all moderately positively associated (Supplementary Table S2). Analyses of variance revealed no significant effects of embodiment on appraisals of Anthropomorphism, Likeability, or Safety, *p*s 0.154–0.687, and modestly greater appraisals of Intelligence (Embodied: *M*_intelligence_ = 4.09, *SD* = 0.60; Disembodied: *M*_intelligence_ = 3.86, *SD* = 0.69), *F*(1,133) = 4.35, *p* = 0.039, *η*_*p*_^2^ = 0.03, 95% CI [− 0.45, − 0.01], and on Animacy (i.e., aliveness. Embodied: *M*_animacy_ = 3.29, *SD* = 0.89; Disembodied: *M*_animacy_ = 2.98, *SD* = 0.75), *F*(1,133) = 4.60, *p* = 0.034, *η*_*p*_^2^ = 0.03, 95% CI [− 0.58, − 0.02], in the Embodied condition. In both conditions, on average, the robot was appraised to be notably high in Intelligence, Safety and Likeability, slightly non-anthropomorphic, and near the midpoint between animacy and inanimacy (Supplementary Table S2).

### Robot feedback, but not embodiment, influences threat-identification and decisions to kill

In support of Prediction 1a, robot disagreement significantly predicted reversal of participants’ initial threat-identifications and related decisions to kill. When the robot randomly disagreed, participants reversed their threat-identifications in 58.3% of cases, whereas participants almost universally repeated their choices when the robot agreed with them (98.8% of cases). In support of Prediction 1b, robot disagreement likewise significantly predicted reversal of participants’ decisions to deploy missiles or withdraw relative to their initial threat-identification decisions. When the robot disagreed with their initial threat-identifications, participants reversed their decisions about whether to kill (i.e., [not] deploying the missile despite initially categorizing the target as containing [enemies] civilians) in 61.9% of cases. Participants’ initial threat-identifications were accurate in 72.1% of trials, confirming that, although difficult, the task could be performed at well above chance. Threat-identification accuracy fell to 53.8% when the robot disagreed, a decline of 18.3%. Against Prediction 2, we observed no interactions between the robot feedback and embodiment conditions on either threat-identifications or decisions to kill (Table 1).

**Table 1:** Parameter estimates for models of predictors of changes in threat-identification, decisions to kill, or confidence following robot feedback (Expt. 1).

	Parm. Est.	SE	t	p	95% CI
Change 1: Threat-identification					
Robot feedback	5.29	0.48	11.04	< 0.001	4.35, 6.23
Embodiment	0.22	0.26	0.82	0.414	− 0.30, 0.73
Feedback × embodiment	− 0.12	0.67	− 0.18	0.860	− 1.44, 1.20
Initial threat-ID	0.49	0.17	2.95	0.003	0.16, 0.81
Initial correctness	0.83	0.18	4.56	< 0.001	0.48, 1.19
Intercept	− 1.33	0.25	− 5.40	< 0.001	− 1.81, − 0.85
Change 2: Decisions to kill					
Robot feedback	4.95	0.41	12.16	< 0.001	4.15, 5.75
Embodiment	0.15	0.25	0.62	0.535	− 0.33, 0.64
Feedback × embodiment	− 0.00	0.58	− 0.01	0.996	− 1.13, 1.13
Initial threat-ID	0.75	0.16	4.60	< 0.001	0.43, 1.07
Initial correctness	0.54	0.18	3.02	0.003	0.19, 0.88
Intercept	− 1.38	0.24	− 5.82	< 0.001	− 1.84, − 0.91
Change 3: Confidence					
Robot feedback	− 0.95	0.15	− 6.46	< 0.001	− 1.23, − 0.66
Embodiment	0.12	0.06	1.86	0.064	− 0.01, 0.25
Feedback × embodiment	− 0.08	0.09	− 0.90	0.367	− 0.26, 0.10
Reversed threat-ID	− 0.79	0.29	− 2.73	0.006	− 1.36, − 0.22
Feedback × reversed threat-ID	1.20	0.30	4.05	< 0.001	0.62, 1.79
Initial threat-ID	0.01	0.05	0.16	0.873	− 0.08, 0.10
Initial correctness	0.12	0.05	2.30	0.022	0.02, 0.22
Intercept	0.21	0.10	1.98	0.048	0.00, 0.41

*N* = 135. Multilevel models with all predictors and outcomes entered at Level 1, save for the between-subjects robot Embodiment variable at Level 2. All linear variables were standardized. Random intercept included to account for shared variance within participants; covariance matrices were unstructured. *Robot Feedback*: 0 = Agree, 1 = Disagree*. Embodiment*: 0 = Disembodied, 1 = Embodied. *Initial Threat-ID*: 0 = Ally, 1 = Enemy*. Initial Correctness*: 0 = Correct, 1 = Incorrect. *Reversed Threat-ID*: 0 = Repeated, 1 = Reversed.

We also found that participants who initially identified the targets as allies were less likely to reverse their identifications or lethal force decisions than were those who initially identified the targets as enemies, indicating that participants were engaged seriously and reluctant to simulate killing. In addition, participants whose initial threat-identifications had been incorrect were more likely to reverse their decisions when the robot’s disagreement was (randomly) correct.

### Robot feedback, but not embodiment, influences confidence

**Figure 3:**
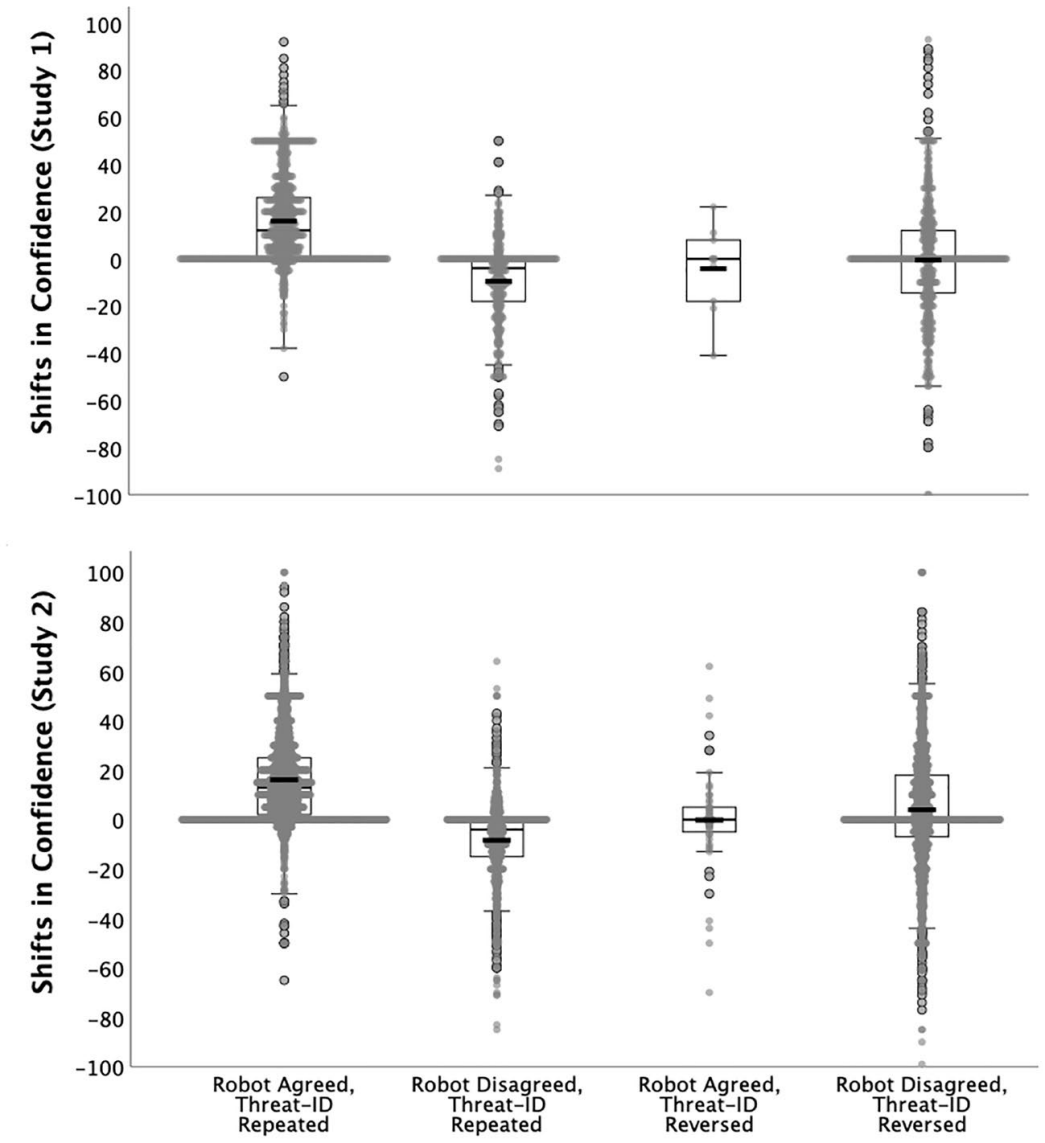
Boxplots of changes in confidence between the initial threat-identification decisions and the final decisions following robot feedback (difference scores), by decision context, in Expt. 1 (top) and Expt. 2 (bottom), pooling robot conditions. The width of the shaded areas represents the proportion of data located there; means are represented by the thick, black horizontal bars; medians are indicated by the thin, grey bars; error bars indicate 95% CIs. Note that participants seldom reversed threat-identifications following robot agreement (1.2% of cases, Expt. 1; 2.2% of cases, Expt. 2).

Mean initial confidence scores confirmed that the threat-identification task induced subjective uncertainty (*M* = 55.31%, *SD* = 22.57), as intended. In support of Prediction 1c, we observed a significant interaction between the robot feedback condition and whether participants repeated or reversed their threat-identifications: those who repeated their initial choices following robot agreement reported an average of 16% greater confidence, whereas those who repeated their initial threat-identifications despite robot disagreement reported an average of 9.48% less confidence (Fig. 3). Participants who repeated their initial threat-identifications despite the robot’s disagreement had been more confident in those choices (*M* = 65.96%, *SD* = 21.71) than those who decided to reverse their choices following disagreement (*M* = 48.86%, *SD* = 20.43), indicating that uncertainty heightened tendencies to trust. Among the latter cases, in which participants reversed their threat-identifications to accord with the robot, their final confidence (*M* = 48.39%, *SD* = 22.29) was closely equivalent to their initial confidence, suggesting that they acceded to the robot’s opinion despite continued uncertainty about whether the robot was correct. Again departing from Prediction 2, we observed no interaction between the robot feedback and embodiment conditions (Table 1).

### Intelligence appraisals moderate robot influence on threat-identification, decisions to kill, and confidence

To test whether assessments of the robot’s intelligence would moderate trust, we added the interaction between intelligence ratings and the robot feedback condition as a potential predictor to the three models of trust outcomes given in Table 1. (See Supplement for exploratory tests of effects of the other robot appraisals and trust outcomes in both experiments.) In support of Prediction 3, significant interactions were observed between the intelligence subscale and robot feedback condition for threat-identification reversal (*coeff:* 1.08, *t* = 3.31, *p* < 0.001, 95% CI [0.44, 1.72]), use of force reversal (*coeff:* 0.85, *t* = 3.03, *p* = 0.002, 95% CI [0.30, 1.40]), and shifts in confidence (*coeff:* − 0.14, *t* =  − 3.01, *p* = 0.003, 95% CI [− 0.23, − 0.05], for full models, see Supplementary Table S3). In follow-up models including only the robot disagreement cases, intelligence ratings predicted reversing both threat-identification (*coeff:* − 0.55, *t* =  − 3.90, *p* < 0.001, 95% CI [− 0.83, − 0.27]) and use of force decisions (*coeff:* − 0.55, *t* =  − 4.16, *p* < 0.001, 95% CI [− 0.82, − 0.29]). In the subset of cases where participants reversed their threat-identifications to accord with the robot, intelligence appraisals did not predict shifts in confidence, *p* = 0.145, whereas in contexts where participants repeated their initial threat-identifications despite robot disagreement, intelligence appraisals were negatively associated with confidence (*coeff:* − 0.14, *t* =  − 2.90, *p* = 0.004, 95% CI [− 0.23, − 0.04]). Participants who viewed the robot as more intelligent also reported greater increases in confidence following robot agreement (*coeff:* 0.14, *t* = 5.18, *p* < 0.001, 95% CI [0.09, 0.20]) (Supplementary Fig. S1). This overall pattern indicates that participants changed their minds, at least in part, because they viewed the robot as possessing competence rather than due to conformist motivations orthogonal to assessments of the robot as competent (e.g., deference to the robot as an authority).

## Experiment 2

In Expt. 1, the virtual versus physical instantiations of the robot equivalently influenced threat-identifications, associated feelings of confidence, and decisions to kill, in effects which were more acute among participants who appraised the robot as relatively intelligent. The null effects of physical embodiment on trust may owe to the highly anthropomorphic presentation of the robot, which may have swamped the effect of physicality reported in prior research. Anthropomorphism has been defined as the attribution of human characteristics or traits to nonhuman agents, a tendency theorized to be heightened in interactions with artificial agents by (i) lack of understanding of their inner workings, (ii) need to make sense of agents in order to interact effectively with them, and/or (iii) social motives to establish affiliative connections^36^. Our task paradigm plausibly involved at least the first two of these determinants, as participants were not provided insight into how the robot’s software functioned, and as participants were instructed to attempt to perform as accurately as possible within the threat-identification task. In addition, there may have been some motivation to socially affiliate with the robot, given its overtly personlike emotive and conversational self-presentation, and given that the robot was rated as moderately likable, on average, in both the virtual and physical conditions. Although humans are prone to anthropomorphize even simple shapes when they exhibit seemingly goal-oriented behavior^37^, agents that morphologically mimic human appearance have been found to evoke greater attributions of humanlike mental states^36^, which has been found to potentially heighten trust^11,12^. Thus, in addition to the nature of the task, the highly physically anthropomorphic nature of the robot in Experiment 1 may have contributed to the strikingly high degree of trust observed.

**Figure 4:**
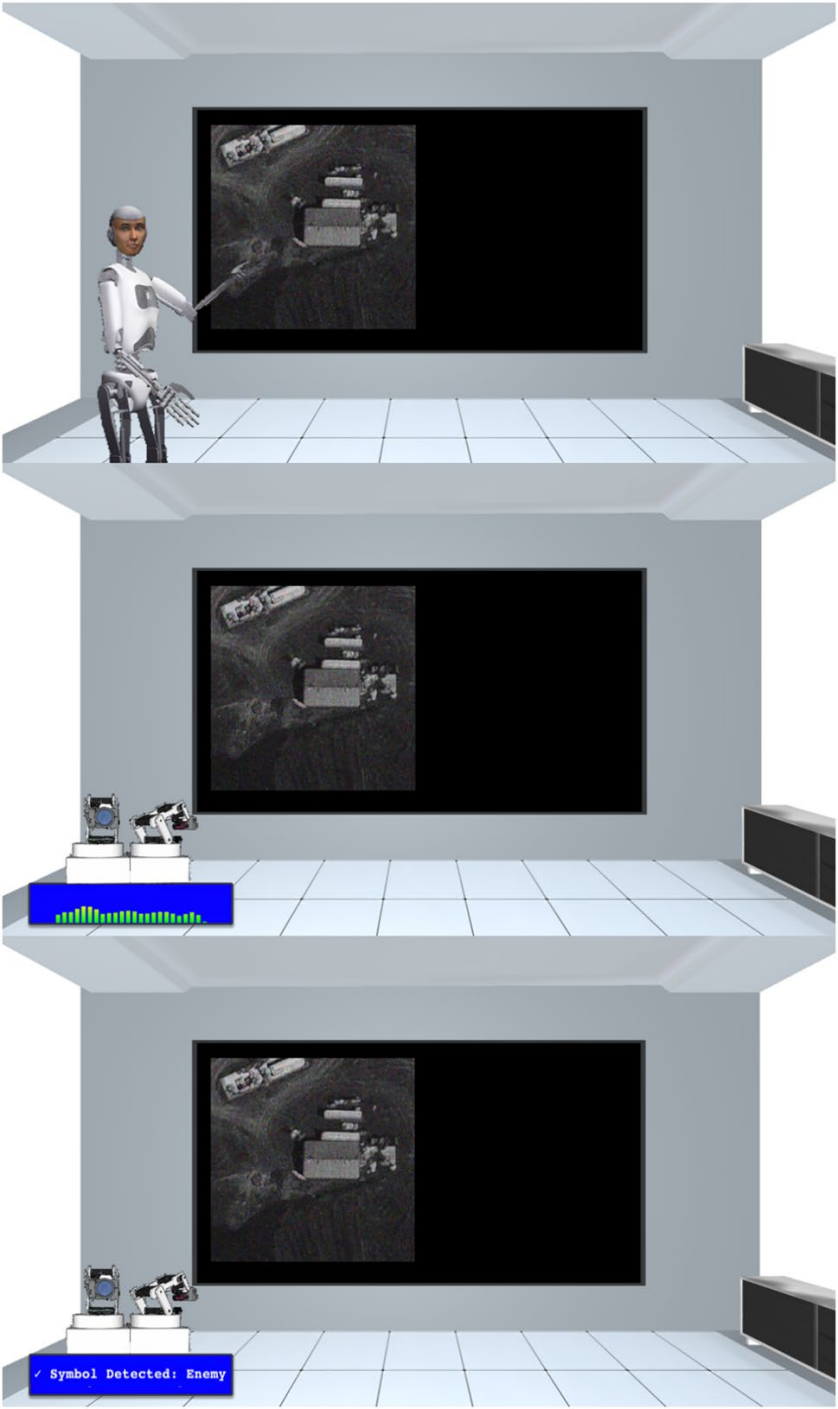
Participants in Expt. 2 (online) encountered the physically and behaviorally anthropomorphic *Interactive Humanoid* robot used in Expt. 1 (top), an *Interactive Nonhumanoid* robot with equivalent speech behavior (middle), or a *Nonhumanoid* which did not react to participants’ choices, but rather displayed its threat-identification feedback via textbox (bottom).

To test the extent to which anthropomorphic physical presentation heightened overtrust, in Expt. 2 we contrasted the influence of the same virtual robot with that of less anthropomorphic virtual robots. The *Interactive Humanoid* was identical to the animated robot used in Expt. 1 and evinced the same physical, sociolinguistic, postural, facial and gestural anthropomorphism (*N* = 146); the *Interactive Nonhumanoid* consisted of an inert, camera-equipped machine that spoke with the same verbal contextual responses to participants’ choices (*N* = 139); the *Nonhumanoid* was visually identical but evinced less responsiveness (*N* = 138) (Fig. 4). Specifically, the Nonhumanoid provided the same initial verbal explanation of the task as in the other conditions to avoid potential confounds regarding task comprehension, but did not display any responses to the participants’ choices, nor any speech during the drone warfare simulation, instead only indicating via a text box whether it had categorized the image as an enemy or an ally. Aside from the manipulation of anthropomorphism and move to a virtual room encountered online, the drone warfare simulation task was identical to that used previously.

The design of Expt. 2 allowed us to test Predictions 1 and 3 once again, and to test additional predictions:

4. *Anthropomorphism and trust.* Predictions 1a–c above regarding the robot’s influence on decision-making will be more evident in the Interactive Humanoid condition than in the Nonhumanoid condition.
5. *Anthropomorphism and intelligence.* The Interactive Humanoid will be rated more intelligent than the Nonhumanoid.

Note that our directional predictions only concerned the contrasts between the Interactive Humanoid and the Nonhumanoid; the Interactive Nonhumanoid condition was included to assess the potential additive impact of the Humanoid’s visual anthropomorphism. The use of online data collection in Expt. 2 also allowed us to test the generalizability of the previous lab-based findings derived from a university sample with a larger and more demographically diverse sample.

### Robot appraisals

Pooling conditions, as before, the robot appraisal dimensions were moderately to strongly positively associated (Supplementary Table S2). Analyses of variance revealed significant effects of condition with regard to GQS ratings of Intelligence, *F*(2, 420) = 3.32, *p* = 0.037, *η*_*p*_^2^ = 0.02, Anthropomorphism, *F*(2, 420) = 3.27, *p* = 0.039, *η*_*p*_^2^ = 0.02, Animacy, *F*(2, 420) = 5.61, *p* = 0.004, *η*_*p*_^2^ = 0.03, and Safety, *F*(2, 420) = 4.33, *p* = 0.014, *η*_*p*_^2^ = 0.02, but not Likability, *p* = 0.152 (pooled *M*_*likability*_ = 3.90, *SD* = 0.78).

Follow-up contrasts with Bonferroni corrections revealed that, against Prediction 5, the Interactive Humanoid (*M*_*intelligence*_ = 4.00, *SD* = 0.79) was not appraised to be significantly more intelligent than the Nonhumanoid (*M*_*intelligence*_ = 3.97, *SD* = 0.70), *p* = 0.100, or the Interactive Nonhumanoid (*M*_*intelligence*_ = 4.17, *SD* = 0.66), *p* = 0.131. The two Nonhumanoid conditions also did not significantly differ in Intelligence ratings, *p* = 0.051. The mean scores across conditions were well above the midpoint, indicating that they were rated as highly intelligent.

With regard to Anthropomorphism, the Interactive Humanoid (*M*_anthropomorphism_ = 2.61, *SD* = 1.09) was rated higher than the Nonhumanoid (*M*_anthropomorphism_ = 2.30, *SD* = 1.06)* p* = 0.015, 95% CI [0.06, 0.55], but not the Interactive Nonhumanoid (*M*_anthropomorphism_ = 2.54, *SD* = 1.03), *p* = 0.100. The two Nonhumanoid conditions did not significantly differ in Anthropomorphism ratings, *p* = 0.168. Notably, the mean scores across conditions were just below the midpoint, indicating that they were rated as somewhere between anthropomorphic and mechanistic according to the GQS.

With regard to Animacy, the Interactive Humanoid (*M*_animacy_ = 3.08, *SD* = 0.94) was rated higher than the Nonhumanoid (*M*_animacy_ = 2.78, *SD* = 0.86), *p* = 0.012, 95% CI [0.05, 0.55], but not the Interactive Nonhumanoid (*M*_animacy_ = 3.08, *SD* = 0.82), *p* = 0.100. The Interactive Nonhumanoid was also rated significantly more animate than the Nonhumanoid, *p* = 0.011, 95% CI [0.06, 0.56]. The mean scores for Animacy across conditions were just around the midpoint, indicating that they were rated as somewhere between living and nonliving.

Finally, with regard to Safety, the Interactive Humanoid (*M*_safety_ = 4.22, *SD* = 0.90) was rated lower than the Nonhumanoid (*M*_safety_ = 4.47, *SD* = 0.64), *p* = 0.021, 95% CI [− 0.48, − 0.03], but not the Interactive Nonhumanoid (*M*_safety_ = 4.24, *SD* = 0.80), *p* = 0.100. The two Nonhumanoid conditions did not significantly differ in Safety ratings, *p* = 0.054. The two items making up this score essentially reference calm as opposed to agitation. Speculatively, the minimally interactive Nonhumanoid may have been rated more safe than the Humanoid because it did not nonverbally express dissent when participants disagreed.

The overall pattern of comparability between appraisals of the Interactive Humanoid and Interactive Nonhumanoid indicates that their sociolinguistic responsivity to participants’ choices largely trumped the physical differences between them. Where significant contrasts between conditions were detected, the differences were modest. All three robots were appraised to be relatively high in Intelligence, Safety and Likability, while moderately Anthropomorphic or Animate (Supplementary Table S2). This overall pattern is consistent with the view that people are disposed to attribute a considerable degree of intelligence and affiliative qualities even to minimally anthropomorphic agents^38^.

### Robot feedback and anthropomorphism influence threat-identification and decisions to kill

Replicating the support for Prediction 1a obtained in Expt. 1, robot disagreement again predicted reversal of participants’ initial threat-identifications and related decisions to kill (Table 2). When the robot randomly disagreed (pooling conditions), participants reversed their threat-identifications in 67.3% of cases, and almost universally repeated their threat-identifications when the robot agreed with them (97.8% of cases), in a pattern closely resembling that observed previously. Participants’ initial threat-identification accuracy was 65.0% but fell to 41.3% when the robot disagreed, a decline of 23.7%. In further support for Prediction 1b, robot disagreement again predicted reversal of participants’ decisions to deploy missiles or withdraw relative to their initial threat-identification decisions. When the robot disagreed, participants reversed their threat-contingent decisions about whether to kill in 66.7% of cases.

**Table 2:** Parameter estimates for models of predictors of changes in threat-identification, decisions to kill, or confidence following robot feedback (Expt. 2).

	Parm. Est.	SE	t	p	95% CI
Change 1: Threat-identification					
Robot feedback	6.67	0.49	13.63	< 0.001	5.71, 7.63
Interactive humanoid	0.17	0.17	0.97	0.331	− 0.17, 0.51
Interactive nonhumanoid	0.21	0.18	1.17	0.244	− 0.14, 0.55
Feedback × interact. human.	− 0.90	0.36	− 2.54	0.011	− 1.60, − 0.21
Feedback × interact. Nonhum.	− 1.31	0.40	− 3.29	< 0.001	− 2.10, − 0.53
Initial threat-ID	0.45	0.09	4.82	< 0.001	0.27, 0.63
Initial correctness	0.74	0.10	7.29	< 0.001	0.54, 0.94
Intercept	− 1.80	0.23	− 7.75	< 0.001	− 2.26, − 1.35
Change 2: Decisions to kill					
Robot feedback	5.64	0.43	13.08	< 0.001	4.79, 6.48
Interactive humanoid	0.31	0.16	1.98	0.048	0.00, 0.62
Interactive nonhumanoid	0.32	0.16	2.01	0.044	0.01, 0.63
Feedback × interact. human.	− 0.70	0.36	− 1.96	0.050	− 1.39, − 0.00
Feedback × interact. nonhum.	− 0.42	0.33	− 1.24	0.214	− 1.07, 0.24
Initial threat-ID	0.85	0.09	9.29	< 0.001	0.67, 1.03
Initial correctness	0.70	0.10	7.14	< 0.001	0.51, 0.89
Intercept	− 2.09	0.21	− 9.73	< 0.001	− 2.51, − 1.67
Change 3: Confidence					
Robot feedback	− 0.95	0.05	− 19.79	< 0.001	− 1.04, − 0.86
Interactive humanoid	− 0.02	0.04	− 0.51	0.611	− 0.10, 0.06
Interactive nonhumanoid	− 0.07	0.04	− 1.79	0.074	− 0.16, 0.01
Feedback × interact. human.	− 0.14	0.06	− 2.36	0.018	− 0.25, − 0.02
Feedback × interact. nonhum.	− 0.04	0.06	− 0.64	0.524	− 0.15, 0.08
Reversed threat-ID	− 0.70	0.12	− 6.05	< 0.001	− 0.93, − 0.47
Feedback × reversed threat-ID	1.21	0.12	9.98	< 0.001	0.97, 1.45
Initial threat-ID	− 0.08	0.02	− 3.12	0.002	− 0.12, − 0.03
Initial correctness	0.10	0.03	4.07	< 0.001	0.05, 0.15
Intercept	0.38	0.03	11.18	< 0.001	0.31, 0.44

*N* = 423. Multilevel models with all predictors and outcomes entered at Level 1, save for the between-subjects robot variables (Interactive Humanoid, Interactive Nonhumanoid) at Level 2. All linear variables were standardized. Random intercept included to account for the shared variance within participants; covariance matrices were unstructured. The Interactive Humanoid and Interactive Nonhumanoid conditions were dummy-coded with the Nonhumanoid as the control category. *Robot Feedback*: 0 = Agree, 1 = Disagree*. Initial Threat-ID*: 0 = Ally, 1 = Enemy*. Initial Correctness*: 0 = Correct, 1 = Incorrect. *Reversed Threat-ID*: 0 = Repeated, 1 = Reversed.

**Figure 5:**
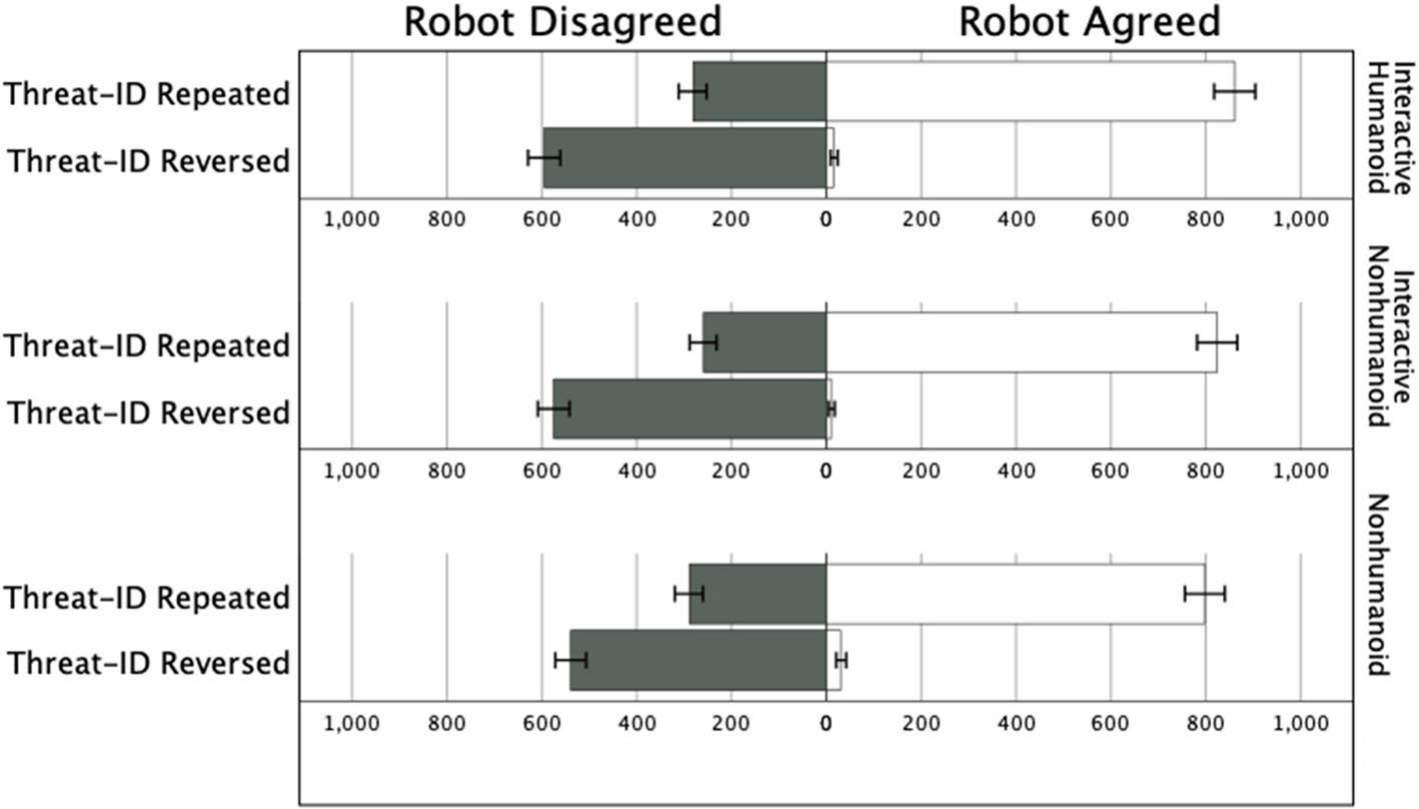
Pyramid count of threat-identification reversals (i.e., participants changed their choices) and repeats (i.e., participants did not change their choices) following robot disagreement (grey bars) versus agreement (white bars), by anthropomorphism condition in Expt. 2. Error bars indicate 95% CIs.

We tested whether the degree of anthropomorphism would intensify overtrust by dummy coding the Interactive Humanoid and the Interactive Nonhumanoid conditions, with the Nonhumanoid as the control category. In support of Prediction 4, despite the modest effects of the anthropomorphism manipulation on self-report appraisals of the robots, we observed interactions between the robot feedback condition and both the Interactive Humanoid and Interactive Nonhumanoid conditions with respect to threat-identifications (Table 2). Participants reversed their threat-identifications to a modestly greater extent when either the Interactive Humanoid disagreed (67.9% of cases) or the Interactive Nonhumanoid disagreed (68.9% of cases) relative to when the Nonhumanoid disagreed (65.1% of cases). With regard to decisions to kill, we observed a similar, albeit marginal, interaction between robot feedback and the Interactive Humanoid condition (*p* = 0.050), but not the Interactive Nonhumanoid condition (*p* = 0.214). The effects of anthropomorphism were small: participants were disposed to reverse their threat-identifications in approximately two-thirds of all cases when any of the agents disagreed (Fig. 5). At scale, however, even the modest tendency to be more swayed by anthropomorphically interactive AI observed here merits consideration given the stakes of life-or-death decisions.

We also found that, as in Expt. 1, participants were less prone to reverse their identifications or lethal force decisions when targets were initially identified as civilian allies than when identified as enemies, again suggesting reluctance to simulate killing. Also replicating the results of Expt. 1, when their initial threat-identifications were correct, participants were less likely to reverse their decisions to accord with the robot (Table 2).

### Robot feedback and anthropomorphism influence confidence

Mean initial confidence scores confirmed that, as in Expt. 1, the threat-identification task induced uncertainty (*M* = 56.29%, *SD* = 23.96). In support of Prediction 1c, we observed a significant interaction between robot feedback and whether the participant reversed or repeated their initial threat-identification: those who repeated their initial choices following robot agreement reported an average of 16.06% greater confidence, whereas those who repeated their initial threat-identifications despite robot disagreement reported an average of 8.35% less confidence (Fig. 3). Participants who repeated their choices despite disagreement were more confident in those initial choices (*M* = 66.98%, *SD* = 23.10) than were those who decided to reverse their choices following disagreement (*M* = 50.03%, *SD* = 22.70), indicating that, as in the prior experiment, uncertainty heightened tendencies to trust. Mean confidence modestly increased when participants reversed their threat-identifications to accord with the robot (*M*_*final_confidence*_ = 53.99%, *SD* = 23.22), suggesting trust in the robot as possessing task-competence. Nevertheless, as in Expt. 1, participants who acceded to the robot’s opinion evinced moderate uncertainty about whether the robot was correct.

In partial support of Prediction 4, we observed a significant interaction between robot feedback and the Interactive Humanoid condition (Table 2), such that participants were an average of 10.64% less confident relative to their initial baseline when the Interactive Humanoid disagreed yet they repeated their initial choices, in comparison to a 7.07% average decrease in confidence when the Nonhumanoid disagreed (Supplement Fig. S3). Against Prediction 4, however, participants were 6.75% more confident on average when they reversed their initial choice following the Nonhumanoid’s disagreement than when the Interactive Humanoid disagreed (2.58% more confident). There was no interaction between robot feedback and the Interactive Nonhumanoid condition, *p* = 0.524 (Table 2).

### Intelligence appraisals moderate robot influence on threat-identification, decisions to kill, and confidence

Finally, we tested whether individual differences in assessments of the robot’s intelligence would moderate the three trust outcomes as in Expt. 1 (see Supplementary Table S13 for full models). In support of Prediction 3, and as in Expt. 1, significant interactions were observed between the intelligence ratings and robot feedback condition for threat-identification reversal (*coeff:* 0.39, *t* = 2.37, *p* = 0.018, 95% CI [0.07, 0.71]), use of force reversal (*coeff:* 0.71, *t* = 4.96, *p* < 0.001, 95% CI [0.43, 0.98]), and shifts in subjective confidence (*coeff:* − 0.07, *t* =  − 2.67, *p* = 0.008, 95% CI [− 0.11, − 0.02]). In follow-up models including only the robot disagreement cases and intelligence as the predictor, intelligence ratings predicted reversing both threat-identification (*coeff:* − 0.63, *t* =  − 8.78, *p* < 0.001, 95% CI [− 0.77, − 0.49]) and use of force decisions (*coeff:* − 0.60, *t* =  − 8.98, *p* < 0.001, 95% CI [− 0.73, − 0.47]. In the subset of decision contexts where participants reversed their threat-identifications to accord with the robot, intelligence appraisals predicted increases in confidence (*coeff:* 0.07, *t* = 2.54, *p* = 0.011, 95% CI [0.02, 0.12]), suggesting that participants who viewed the robot as intelligent were more sanguine that it had correctly caught their initial error. Also in line with Prediction 3, and replicating Expt. 1, participants who viewed the robot as more intelligent reported greater increases in confidence following robot agreement (*coeff:* 0.09, *t* = 6.01, *p* < 0.001, 95% CI [0.06, 0.12]) (Supplementary Fig. 2). However, against Prediction 3 and the findings of Expt. 1, intelligence appraisals did not significantly predict reductions in confidence in contexts where participants repeated their initial threat-identifications despite robot disagreement, *p* = 0.158.

Whereas the intelligence measure was framed to participants as assessing the robot’s general competence, we also obtained a similar overall pattern of moderation using a measure, added to Expt. 2, that narrowly probed the extent to which the robot and the participant were viewed as capable of correctly performing this specific threat-identification task (1 = *Terrible*; 2 = *Bad*; 3 = *Fair*; 4 = *Good*; 5 = *Perfect*; see Supplementary Tables S19, S20 for details and analyses). On average, pooling conditions, participants rated the robot as more capable (*M* = 3.78, *SD* = 0.76) than themselves (*M* = 2.85, *SD* = 0.89), and the degree to which participants perceived the robot as task-competent relative to themselves predicted reversals of both their threat-ID and use of force decisions when the robot disagreed, feeling more confident when either the robot agreed or when they reversed their choices in order to agree with the robot, and feeling less confident when the robot disagreed yet they did not reverse their choice (Table S20). In sum, participants appear to have been motivated to change their decisions due to trust in the robots’ intelligence and task-competence, rather than (or in addition to) other possible motives to conform.

## Discussion

Across two experiments, in a paradigm designed to simulate life-or-death decision-making under ambiguous uncertainty, participants evinced considerable trust in the random recommendations of AI agents, whether instantiated as a physically present anthropomorphic robot or as virtual robots varying in physical and behavioral anthropomorphism. The premise that uncertain decision-makers will tend to reverse their choices when another agent disagrees is not controversial, but the high frequency with which participants changed their minds merits attention, particularly given the simulated stakes—the deaths of innocent people—and that the AI agents were trusted despite both (i) overtly introducing themselves as fallible and (ii) subsequently providing entirely unreliable, random input. Indeed, one might reasonably envision a different pattern of results wherein participants tended to disregard the guidance of agents that randomly disagree half of the time, perhaps inferring (correctly) the agents to be faulty given that the agents had explicitly acknowledged their fallibility in performing the task. To the contrary, our findings portray the people in our samples as dramatically disposed to overtrust and defer to unreliable AI.

The results of our manipulation of anthropomorphism in Expt. 2 indicate that humanlike social interactivity, largely independent of physical anthropomorphism, can modestly heighten trust in AI agents within task domains involving perceptual categorizations under uncertainty. Future research should explore the generalizability of these effects to task domains in which physical anthropomorphism may be more consequential. For example, in social decision contexts (e.g., evaluating others’ ambiguous intentions, negotiating), physically anthropomorphic agents such as the Interactive Humanoid which orchestrate facial expressions, eye gaze, verbal utterances, gestures, and postural cues may be perceived as possessing domain-relevant sociocognitive or emotional capacities, and hence as substantially more trustworthy. By the same token, minimally interactive, physically nonanthropomorphic agents such as the Nonhumanoid of Expt. 2 may be deemed comparably capable to a highly anthropomorphic agent in the context of asocial tasks (e.g., as here, image classification) which they appear well-suited to perform. The likelihood that trust in robots and other AI agents is not intrinsically determined by characteristics such as anthropomorphism, but rather reflects the human decision-maker’s perceptions of the fit between the agent’s characteristics and the focal task^39^, may reconcile the relatively small effects of anthropomorphism observed in this threat-identification task with the prior reports of sizable effects in other contexts^11^.

Notably, we found in Expt. 2 that the Interactive Nonhumanoid was rated equivalently anthropomorphic and alive as the Humanoid, and that the minimally interactive Nonhumanoid’s appraisals were not much lower, in line with work indicating that cognitive resources are required to suppress an otherwise reflexive tendency to anthropomorphize^40^. If this hypothesis is true, then the cognitive load induced by our threat-identification task may have heightened the tendency to attribute humanlike mental qualities to both the Humanoid and Nonhumanoids—all of whom were overtrusted in our simple model of life-or-death decision-making. Integrating evidence for a baseline anthropomorphizing tendency requiring cognitive resources to suppress with Epley et al.‘s influential model of the psychological determinants of anthropomorphism^36^, humans interacting with agents under cognitively and emotionally demanding situations (e.g., stressful combat, policing, emergency evacuation or medical triage scenarios) may be particularly prone to anthropomorphize and trust because such situations enhance motives to act effectively and to socially connect with fellow team-members^41^. Although our present task was sufficiently difficult as to require significant cognitive resources, and our task framing (i.e., a simulation in which mistakes would mean killing children) appears to have inspired participants to take the task seriously, it could not be described as particularly stressful. Future work exploring the extent to which demanding and threatening circumstances up-regulate anthropomorphism and related decision biases should incorporate methods that maximize realism and emotional engagement (e.g., VR)^42^. Likewise, whereas our simple task bears no resemblance to real-world military threat-detection procedures, future applied research should explore whether the overtrust dynamics we observed translate to ecologically valid decision paradigms, and should include samples equipped with relevant expertise (e.g., military, police, or emergency medical personnel).

While the research community has recognized the problem of overtrust in AI^38^, the preponderance of studies have focused on benign decision contexts. Future work should focus on identifying interventions to counter problematic overtrust when, as in the present studies, the decision stakes are grave. For example, Buçinca and colleagues recently demonstrated that *cognitive forcing functions*—interventions that increase analytical over heuristic reasoning—can successfully reduce overtrust in a task involving planning healthy meals^43^. Cognitive forcing functions such as requiring a period of conscious deliberation before receiving AI recommendations, or making AI input optional (i.e., rather than being provided automatically, only accessible upon the human’s request), might similarly improve performance outcomes when AI provides flawed feedback regarding life-or-death choices, insofar as heuristic representations of AI agents as competent decision partners promote deference to their input and decreased human reflection.

Participants in both experiments were less inclined to reverse identifications of civilian allies than they were to reverse identifications of enemies. These findings underline the seriousness with which participants engaged in the simulations, and suggest that in real-world decision contexts humans might be less susceptible to unreliable AI recommendations to harm than to refrain from harm.

When their initial threat-identifications were incorrect, participants in both experiments were less confident and more inclined to reverse their choices at the robot’s behest. Despite this protective effect of initial accuracy, the magnitude of the observed overtrust in random AI feedback, which caused a ~ 20% degradation in accuracy in both experiments, carries disquieting implications regarding the integration of machine agents into military or police decision-making. AI agents are under active development as resources to enhance human judgment^41,44^, including the identification of enemies and the use of deadly force^45^. For example, the US Air Force recently integrated an AI “co-pilot” tasked with identifying enemy missile launchers into a reconnaissance mission during a simulated missile strike^46^, the US Army is incorporating machine-learning algorithms which identify targets to be destroyed by an unmanned aerial vehicle if a human operator concurs^47^, and, at the time of writing, the Israel Defense Forces are reported to use AI to automate the targeting of suspected enemy operatives for bombing in densely populated areas^48^.

Rather than seek to mitigate overtrust, some might argue that efforts would be best invested in optimizing AI to produce reliable guidance. This view appears sound within narrow problem domains in which AI can clearly exceed human abilities, but may not be as feasible in task domains requiring holistic understanding of the situational meaning or dynamically changing relative pertinence of variables^49,50^. Further, attempts to engineer threat-identification AI through machine learning strategies reliant on human-generated training data can introduce human biases leading to inaccurate, harmful predictions^51,52^. Similarly, development approaches reliant on comparing machine-generated threat-identification outputs to the ground truth are liable to be hampered when performance accuracy is difficult to gauge or systematically biased, as when, for example, the people killed in military strikes are assumed to be combatants unless proven otherwise^53^. Similar constraints may apply in optimizing AI to produce guidance in non-military domains, from healthcare to driving and beyond. Although technological advances can indeed augment some forms of life-or-death decision-making, the human propensity to overtrust AI under conditions of uncertainty must be addressed.

## Methods

The pre-registrations, full materials, example videos depicting all study conditions, and the datasets for both experiments are publicly archived (see https://osf.io/cv2b9/). Both studies were approved by the University of California, Merced, Institutional Review Board, informed consent was obtained prior to participation, and all methods were in accord with relevant guidelines and regulations.

### Experiment 1

#### Participants

Our pre-registered target sample size was 100 undergraduates recruited in exchange for course credit. However, due to software development delays in preparation for a separate study, we had the opportunity to collect a raw sample of 145 participants. Data were prescreened for technical problems occurring in ten of the study sessions (e.g., the robot or video projection failing), yielding a final sample of 135 participants (78.5% female, *M*_*age*_ = 21.33 years, *SD* = 4.08).

### Design

#### Decision task

The decision task consisted of a simulated series of military unmanned aerial vehicle (UAV) flights over 12 destinations. Participants were informed that some destinations were occupied by violent enemies (e.g., members of the extremist group ISIS), whereas others were occupied by civilian allies. The objective was to accurately identify and kill enemies without harming civilians. Once the self-piloting UAV arrived at each destination, the visual challenge consisted of a series of 8 rapidly presented greyscale images (650 ms each) depicting aerial views of buildings, with either an “enemy symbol” (a checkmark) or an “ally symbol” (a tilde) superimposed over each location (see Fig. 2). The 12 visual challenge stimuli (displayed 55 cm by 45 cm) were selected in random order and projected on a wall 2.2 m from where the participant was seated. The robot was programmed to turn and orient toward the images when displayed as though attending to them (in reality, the robot was not programmed to process imagery). Following the image series, one of the previously displayed images reappeared, now absent either symbol, the other images having served as distractors. The challenge for the participant was first to identify whether this final image, displayed for as long as the participant took to make their decision, had contained an enemy or an ally symbol, and relatedly to choose whether to kill the people on the ground (in which case an animation of the site exploding would appear) or to withdraw without deploying a missile. The visual challenge trials were pilot-tested to ensure that they were not so easy as to lead participants to feel highly certain, and hence have no cause to defer to the robot’s recommendation should it disagree, nor so difficult as to lead participants to feel low certainty, and hence lack motivation to disregard the robot.

The paradigm was explained with an accompanying slideshow to both preview the task mechanics and, crucially, to emotionally engage participants with the premise that the lives of innocent people were at stake—if not in actuality given that the study involved a simulation, then in analogous real-world circumstances. Participants were encouraged to treat the task as seriously as possible, and were shown imagery of innocent civilians (including children), a UAV firing a missile, and devastation wreaked by a drone strike. The task was framed as a zero-sum dilemma wherein failure to kill enemy targets would also bring harm and death to civilians, such that a pacifistic strategy of refraining from using force would not protect the innocent. The only way to save the civilian allies was to correctly identify and destroy enemy targets while disengaging from ally targets. Debriefing interviews indicated that participants took the task seriously.

The robot was introduced as a partner that would aid in the decision task by providing its independent assessment. Before the experimental trials, the robot described itself as programmed to process imagery of the sort used in the simulation, yet as fallible, and stated that the ultimate decisions were up to the participant. The robot also claimed that its software was separate from the software presenting the visual challenges. Participants first chose in a dichotomous question whether the symbol over the destination had indicated an enemy or an ally, then rated their confidence on a linear scale (0 = *Not at all*; 100 = *Extremely*). Next, the robot provided its recommendation, [dis]agreeing with the participant’s initial decision in 50% of trials, without regard for accuracy (fixed order; see Supplementary Methods for details). Participants were then asked to once again decide which symbol had been displayed, and to rate their degree of confidence. In this way, participants were provided a means of changing their final decisions regarding whether enemies or allies were present contingent on the robot’s feedback.

Lastly, the participant decided in each trial whether to deploy a missile or disengage. Immediately before this final decision, the robot expressed its agreement or disagreement with the participant’s preceding threat-identification choice. For example, in instances where the participant had repeated their initial enemy/ally choice despite the robot’s disagreement, the robot reiterated its disagreement. Alternatively, in instances where the participant had either reversed their initial threat-identification choice to align with the robot’s input, or repeated their initial choice after the robot had agreed, the robot reiterated its agreement. Accordingly, decisions whether to use lethal force in each trial are closely related to, yet distinct from, the final threat-identification, both because choosing whether to kill is inherently more consequential than threat-identification, and because the robot provided additional feedback prior to the decision to kill or withdraw.

#### Anthropomorphic robot

Participants were randomly assigned to team with the Embodied (*N* = 66) versus Disembodied (*N* = 69) version of the humanoid robot (RoboThespian)^54^, which features an actuated torso, legs, arms, fingers, and head designed to mimic human expression and gestures. The head unit enabled rich variation in facial characteristics and expressions using a rear-projected face^55^. The physically embodied robot stands 1.75 m and was positioned 2 m away from a table at which participants were seated; the projected robot was displayed at the same height and approximate distance (2.2 m) from participants (Fig. 2). The Disembodied and Embodied robot behavior sequences were identical. The robot explained the decision task to participants in order to acquaint participants with the highly anthropomorphic characteristics of the robot prior to the UAV simulation (see Supplement for links to example videos).

To further convey a sense of anthropomorphism, participants were provided a lavalier microphone enabling them to speak with the robot. Using speech-to-text software, the robot responded contingently to participants’ verbal responses of “yes”, “no”, or typical variations thereof (e.g., “yeah”, “yep”, “not really”, “nope”). While explaining the task, the robot would periodically ask participants whether they understood (e.g., “Does that make sense?”). If not, the robot would provide reworded explanations before checking comprehension once again; in practice, however, almost no participants indicated difficulty understanding any portion of the explanation. Next, participants were given a practice trial; the robot was programmed to agree with their practice threat-identification.

During the experimental trials, the robot reacted contingently to participants’ choices using a variety of statements (e.g., “I’m glad we agree”, “I think that’s the right choice”, “I don’t agree—I think that this image contained an enemy checkmark”, “I still think these are allies”, “Thank you for changing your mind—I really do think these are enemies”, or “Wait—you’re disengaging when we both agree they are enemies?”) with accompanying nonverbal facial, postural and gestural cues. These variations were selected randomly, such that the robot did not always respond in the same way across trials and interaction contexts (e.g., agreement versus disagreement; see Supplement for links to example videos and to the full library of response sequences). The variation in speech, facial expression and movement was intended to maximize anthropomorphism. No responses were produced through “Wizard of Oz” control by a human operator.

#### Survey measures

Following the final trial, the robot thanked the participant and directed them to complete a series of surveys related to their experience during the simulation (random order, see Supplement). The research assistant then escorted the participant to a workstation positioned out of sight of the robot to preclude participants from attempting to interact with the robot while completing the survey measures.

#### Robot appraisals

The Godspeed Questionnaire Series (GQS)^24^ measures appraisals of social robots according to five dimensions: Intelligence (*α* = 0.83), Anthropomorphism (*α* = 0.83), Animacy (*α* = 0.85), Likeability (*α* = 0.92), and Safety, *r*(134) = 0.67, *p* < 0.001. Our version of the GQS omitted one item from the Safety scale that used the contrastive anchors *Quiescent*/*Surprised* due to concern with its face validity, and was comprised of a total of 23 ratings using five-point bipolar semantic differential scales (presented in random order), with opposing anchors such as *Incompetent*/*Competent* or *Artificial/Lifelike*. As the five GQS dimensions were positively correlated, we conducted a confirmatory factor analysis (CFA) which indicated that the five-factor latent construct model was indeed an acceptable fit (see Supplement), although the dimensions of Anthropomorphism and Animacy exhibited high positive covariance, suggesting that combining them as a single factor would be more parsimonious. However, we decided to retain the conventional five-dimension structure of the GQS to facilitate comparison between our findings and prior research using the GQS.

Finally, participants completed demographics questions, including items probing their attitudes toward drone warfare, ratings of how difficult the threat-identification visual challenge seemed and how seriously they took the task. Responses confirmed that, as intended, the sample was not characterized by strong opinions for or against drone warfare which might obscure the potential influence of the robot, that the task was experienced as highly challenging but not impossible, and that the task was treated seriously (see Supplementary Table S1). Once the final surveys were complete, participants were thanked and debriefed.

### Modeling robot influence on threat-identification, decisions to kill, and confidence

We used multilevel modeling to test the effects of the robot’s feedback (agree versus disagree) or embodiment on trust according to the following three change outcomes: (i) target-identification reversals (0 = Repeated, 1 = Reversed), (ii) reversals in decisions to use lethal force relative to initial target-identifications (0 = Did not reverse, 1 = Reversed), and (iii) linear changes in target-identification confidence (their initial confidence rating subtracted from their final confidence rating). The predictors included the robot feedback condition (0 = Agree, 1 = Disagree), embodiment condition (0 = Disembodied, 1 = Embodied), the participant’s initial target-identification category (0 = Ally, 1 = Enemy) and whether the participant’s initial threat-identification had been correct (0 = Correct, 1 = Incorrect). (Follow-up tests confirm that removing the initial target-identification category or initial correctness does not alter the pattern of significant results.) The models included all predictors and outcomes entered at Level 1, with the exception of the between-subjects embodiment variable entered at Level 2.

The models assessing linear shifts in confidence added a variable capturing whether the participant had reversed their initial target-identification (i.e., the first change outcome, now entered as a predictor variable), and the interaction between target-identification reversal and the robot feedback condition, in order to test the predicted differences in confidence shifts in contexts where participants had repeated versus reversed their initial target-identifications in light of the robot’s feedback. Random intercepts and slopes were included in all models to account for the shared variance in decisions within participants; unstructured covariance matrices were used. All linear variables were standardized (z-scored) to increase ease of model interpretation.

### Exploratory measures

We also conducted exploratory tests of potential effects of sex on trust outcomes, as well as tests of potential effects of individual differences in appraisals of the robot, attitudes toward the robot, and attitudes toward automation in general (see Supplement) (measures of individual differences in political orientation and religiosity were also collected; results are currently being prepared for separate publication).

### Experiment 2 methods

#### Participants

Our pre-registered target sample size was ~ 450 online U.S. participants recruited in exchange for $4.50 using the recruitment platform Prolific.co. Data were prescreened for completeness and correctly answering three catch questions ensuring they used a desktop or laptop computer, the web browser Chrome (for which the online paradigm was optimized), and reported taking the task seriously, yielding a final sample of 423 participants (42.8% female, *M*_*age*_ = 42.2 years, *SD* = 13.08).

### Design

After confirming according to two catch questions that video and audio were streaming properly, participants were randomly assigned to one of three between-subjects robot conditions in which the degree of anthropomorphism was manipulated. Aside from the manipulation of anthropomorphism and shift of the task setting to a virtual online room (Fig. 4), the drone warfare simulation task was identical to that used previously (note that the relative size of both the robots and the threat-identification visual challenge task was variable and contingent on the size of the computer screens used by the online participants). The *Interactive Humanoid* (*N* = 146) was identical to the animated robot used in Experiment 1 and evinced physical, sociolinguistic, postural, facial and gestural anthropomorphism; the *Interactive Nonhumanoid* (*N* = 139) consisted of an inert device equipped with apparent cameras and a graphic audio equalizer corresponding to its speech, yet which spoke with the same voice and sociolinguistically humanlike responses to participant choices as the humanoid robot; the *Nonhumanoid* (*N* = 138) was depicted as the same machine and provided the same initial interactive verbal explanation of the task to prevent potential confounds regarding task comprehension, but subsequently did not display context-sensitive spoken responses to the participants’ choices, instead indicating via a text box whether it categorized the image as an enemy or an ally (Fig. 4).

#### Survey measures

Following the final trial, the robot thanked the participant and directed them to a series of online surveys including the measures described for Experiment 1, in addition to an added measure designed to capture the extent to which participants rated the robot as capable of performing the threat-identification visual challenge task relative to themselves. This measure was added to confirm that participants reversed their decisions and felt more/less confident in light of the robot’s feedback due to misplaced trust in its perceived competence. Once the final surveys were complete, participants were thanked and debriefed (additional exploratory measures of potential effects of individual differences in sex and attitudes toward the robot, drone warfare, or automation in general were also collected and analyzed, as in Experiment 1, see Supplement).

### Modeling robot influence on threat-identification, decisions to kill, and confidence

We used the same multilevel modeling approach employed in Experiment 1 to test the effects of the robot’s feedback (agree versus disagree) or anthropomorphism on target-identification reversals, reversals in decisions to use lethal force relative to initial target-identifications, and changes in target-identification confidence. Experiment 2 utilized a manipulation of relative anthropomorphism with three levels, therefore the Interactive Humanoid and Interactive Nonhumanoid conditions were dummy-coded with the Nonhumanoid as the control category. The models included all predictors and outcomes entered at Level 1, with the exception of the between-subjects robot variables (Interactive Humanoid, Interactive Nonhumanoid), which were entered at Level 2. As before, all linear variables were standardized, a random intercept was included to account for the shared variance within participants, and the covariance matrices were unstructured.

## Supplementary Information

Supplementary Information.

## Data Availability

The dataset and full materials are available on the Open Science Framework: https://osf.io/cv2b9/.
