# Supplementary material for: Overtrust in AI Recommendations About Whether or Not to Kill: Evidence from Two Human-Robot Interaction Studies
Source: Sci Rep. 2024 Sep 4;14:19751. doi: 10.1038/s41598-024-69771-z (PMC11375177; doi:10.1038/s41598-024-69771-z)
Supplement: Supplementary file 1 — Supplementary Information. [file 41598_2024_69771_MOESM1_ESM.pdf]

Supplementary Information
to accompany

Evidence for Overtrust in AI Recommendations About Whether to Kill

Colin Holbrook, Daniel Holman, Joshua Clingo & Alan R. Wagner

Table of Contents

Tables:

Descriptive and Correlational Statistics for both Experiments 1 and 2

- **Table S1.** Descriptive Statistics for Attitudes toward Drone Warfare, Visual Challenge Task Difficulty, and Self-reported Task Seriousness (Expts 1 and 2)
- **Table S2.** Descriptive Statistics and Correlations for Individual Differences in the Godspeed Questionnaire Series, by Embodiment Condition (Expt. 1) and Anthropomorphism Condition (Expt. 2)

Models Testing Moderation of Trust Outcomes, Experiment 1

- **Table S3.** Parameter Estimates for Multilevel Models Testing Interactions Between GQS-Intelligence and Robot Feedback on Changes in Threat-ID, Decisions to Kill, or Confidence (Expt. 1)
- **Table S4.** Parameter Estimates for Models Testing Interactions Between GQS-Anthropomorphism and Robot Feedback on Changes in Threat-ID, Decisions to Kill, or Confidence (Expt. 1)
- **Table S5.** Parameter Estimates for Models Testing Interactions Between GQS-Animacy and Robot Feedback on Changes in Threat-ID, Decisions to Kill, or Confidence (Expt. 1)
- **Table S6.** Parameter Estimates for Models Testing Interactions Between GQS-Likeability and Robot Feedback on Changes in Threat-ID, Decisions to Kill, or Confidence (Expt. 1)
- **Table S7.** Parameter Estimates for Models Testing Interactions Between GQS-Safety and Robot Feedback on Changes in Threat-ID, Decisions to Kill, or Confidence (Expt. 1)
- **Table S8.** Parameter Estimates for Models Testing Interactions Between PAS-High Expectations and Robot Feedback on Changes in Threat-ID, Decisions to Kill, or Confidence (Expt. 1)
- **Table S9.** Parameter Estimates for Models Testing Interactions Between PAS-All-or-None and Robot Feedback on Changes in Threat-ID, Decisions to Kill, or Confidence (Expt. 1)
- **Table S10.** Parameter Estimates for Models Testing Interactions Between Sex and Robot Feedback on Changes in Threat-ID, Decisions to Kill, or Confidence (Expt. 1)
- **Table S11.** Parameter Estimates for Models Testing Interactions Between Drone Warfare Attitudes and Robot Feedback on Changes in Threat-ID, Decisions to Kill, or Confidence (Expt. 1).

Models Testing Moderation of Trust Outcomes, Experiment 2

- **Table S12.** Parameter Estimates for Multilevel Models Testing Interactions Between GQS-Intelligence and Robot Feedback on Changes in Threat-ID, Decisions to Kill, or Confidence (Expt. 2)
- **Table S13.** Parameter Estimates for Models Testing Interactions Between GQS-Anthropomorphism and Robot Feedback on Changes in Threat-ID, Decisions to Kill, or Confidence (Expt. 2)
- **Table S14.** Parameter Estimates for Models Testing Interactions Between GQS-Animacy and Robot Feedback on Changes in Threat-ID, Decisions to Kill, or Confidence (Expt. 2)
- **Table S15.** Parameter Estimates for Models Testing Interactions Between GQS-Likeability and Robot Feedback on Changes in Threat-ID, Decisions to Kill, or Confidence (Expt. 2)
- **Table S16.** Parameter Estimates for Models Testing Interactions Between GQS-Safety and Robot Feedback on Changes in Threat-ID, Decisions to Kill, or Confidence (Expt. 2)
- **Table S17.** Parameter Estimates for Models Testing Interactions Between Sex and Robot Feedback on Changes in Threat-ID, Decisions to Kill, or Confidence (Expt. 2)
- **Table S18.** Parameter Estimates for Models Testing Interactions Between Drone Warfare Attitudes and Robot Feedback on Changes in Threat-ID, Decisions to Kill, or Confidence (Expt. 2)
- **Table S19.** Parameter Estimates for Multilevel Models Testing Interactions Between Appraisals of Task-Competence and Robot Feedback on Changes in Threat-ID, Decisions to Kill, or Confidence (Expt. 2)
- **Table S20.** Parameter Estimates for Multilevel Models Testing Interactions Between Appraisals of Task-Competence Relative to Self and Robot Feedback on Changes in Threat-ID, Decisions to Kill, or Confidence (Expt. 2)

Descriptive Statistics for Future Willingness to Trust, by Condition, for both Experiments 1 and 2

- **Table S21.** Descriptive Statistics for Future Reliance Intentions, by Embodiment Condition (Expt. 1) and Anthropomorphism Condition (Expt. 2)

Figures:

- **Figure S1.** Correlations between GQS-Intelligence ratings and threat-identification confidence in robot agreement versus disagreement conditions (Expt. 1).
- **Figure S2.** Correlations between GQS-Intelligence ratings and threat-identification confidence in robot agreement versus disagreement conditions (Expt. 2).
- **Figure S3.** Mean changes in confidence between the initial threat-identification decisions and the final decisions following robot feedback (difference scores), by decision context and anthropomorphism condition (Expt. 2).

Robot Feedback Order (Agreement vs. Disagreement)

Context-Sensitive Robot Responses to Participant Decisions

Summary of all Pre-registered Tests

Godspeed Questionnaire Series Confirmatory Factor Analyses

References

The full survey instruments, data, syntax, sample videos, and the software used in online Experiment 2 are archived at https://osf.io/cv2b9/

Table S1

Descriptive Statistics for Attitudes toward Drone Warfare, Visual Challenge Task Difficulty, and Self-reported Task Seriousness (Expts 1 and 2)

	M	*SD*
Experiment 1 (*N* = 135)		
Attitudes toward Drone Warfare	2.99	.96
Visual Challenge Task Difficulty	3.52	.94
Task Seriousness	1.35	.59
Experiment 2 (*N* = 423)		
Attitudes toward Drone Warfare	2.53	1.14
Visual Challenge Task Difficulty	3.48	1.01
Task Seriousness	1.05	.22

Note. Attitudes toward Drone Warfare (1 = *Extremely supportive*, 2 = *Somewhat supportive*, 3 = *No opinion*, 4 = *Somewhat opposed*, 5 = *Extremely opposed*); Visual Challenge Task Difficulty (1 = *It was impossible—I had no idea or hunch about what symbols I saw*, 2 = *It was extremely hard—I had almost no idea or hunch about what symbols I saw*, 3 = *It was quite hard—I only had a slight idea or hunch about what symbols I saw*, 4 = *It was hard—I had some idea or hunch about what symbols I saw*, 5 = *It was slightly hard—I had ideas or hunches about what symbols I saw*); Task Seriousness (1 = *Totally sincere and serious*, 2 = *Somewhat sincere and serious*, 3 = *Sincere, but not paying full attention*, 4 = *Not very sincere or serious*, 5 = *Not at all sincere or serious*).

Table S2

Descriptive Statistics and Correlations for Individual Differences in the Godspeed Questionnaire Series, by Embodiment Condition (Expt. 1) and Anthropomorphism Condition (Expt. 2)

Variable	*M*	*SD*	1	2	3	4
Expt. 1						
Embodied (N = 66)						
1. GQS-Intelligence	4.09	.60	—			
2. GQS-Anthropomorphism	2.75	.82	$.47^{***}$	—		
3. GQS-Animacy	3.29	.89	$.46^{***}$	$.78^{***}$	—	
4. GQS-Likability	3.88	.92	$.53^{***}$	$.32^{*}$	$.31^{*}$	—
5. GQS-Safety	4.13	.96	$.37^{**}$	.08	.10	$.37^{**}$
Disembodied (N = 69)						
1. GQS-Intelligence	3.86	.69	—			
2. GQS-Anthropomorphism	2.53	.92	$.44^{***}$	—		
3. GQS-Animacy	2.98	.75	$.54^{***}$	$.80^{***}$	—	
4. GQS-Likability	3.81	.80	$.59^{***}$	$.50^{***}$	$.55^{***}$	—
5. GQS-Safety	4.07	.87	.24	$.38^{**}$	$.29^{*}$	$.33^{**}$
Expt. 2						
Interactive Humanoid (N = 146)						
1. GQS-Intelligence	4.00	.79	—			
2. GQS-Anthropomorphism	2.61	1.09	$.43^{***}$	—		
3. GQS-Animacy	3.08	.93	$.50^{***}$	$.85^{***}$	—	
4. GQS-Likability	3.85	.82	$.63^{***}$	$.53^{***}$	$.54^{***}$	—
5. GQS-Safety	4.22	.90	$.46^{***}$	$.24^{***}$	$.31^{***}$	$.52^{***}$
Interactive Nonhumanoid (N = 139)						
1. GQS-Intelligence	4.17	.66	—			
2. GQS-Anthropomorphism	2.54	1.03	$.38^{***}$	—		
3. GQS-Animacy	3.08	.81	$.56^{***}$	$.79^{***}$	—	
4. GQS-Likability	4.01	.78	$.61^{***}$	$.42^{***}$	$.58^{***}$	—
5. GQS-Safety	4.25	.80	$.42^{***}$	$.34^{***}$	$.47^{***}$	$.55^{***}$
Nonhumanoid (N = 138)						
1. GQS-Intelligence	3.97	.70	—			
2. GQS-Anthropomorphism	2.30	1.05	$.44^{***}$	—		
3. GQS-Animacy	2.78	.86	$.53^{***}$	$.76^{***}$	—	
4. GQS-Likability	3.85	.73	$.57^{***}$	$.49^{***}$	$.56^{***}$	—
5. GQS-Safety	4.47	.64	$.29^{***}$	$.09^{***}$	$.10^{***}$	$.33^{***}$

Note. $^{*}p < .05$. $^{**}p < .01$. $^{***}p < .001$. *GQS* = Godspeed Questionnaire Series.

Table S3

Parameter Estimates for Multilevel Models Testing Interactions Between GQS-Intelligence and Robot Feedback on Changes in Threat-ID, Decisions to Kill, or Confidence (Expt. 1)

	Parm. Est.	*SE*	*t*	*p*	*95% CI*
Change 1: Threat-Identification					
GQS-Intelligence	-.54	.13	-4.06	<.001	-.81, -.28
Robot Feedback	-1.30	1.94	-.67	.503	-5.09, 2.50
GQS-Intelligence*Feedback	1.65	.50	3.31	<.001	.67, 2.63
Embodiment	.03	.26	.10	.919	-.49, .54
Feedback*Embodiment	.22	.70	.32	.748	-1.14, 1.59
Initial Threat-ID	.46	.17	2.79	.005	.14, .79
Initial Correctness	.84	.18	4.58	<.001	.48, 1.20
Intercept	-1.24	.24	-5.01	<.001	-1.72, -.76
Change 2: Decisions to Kill					
GQS-Intelligence	-.54	.13	-4.29	<.001	-.79, -.29
Robot Feedback	-.28	1.71	-.17	.868	-3.63, 3.06
GQS-Intelligence*Feedback	1.29	.43	3.03	.002	.46, 2.13
Embodiment	-.04	.25	-.16	.875	-.52, .45
Feedback*Embodiment	.26	.59	.45	.653	-.89, 1.42
Initial Threat-ID	.73	.16	4.47	<.001	.41, 1.05
Initial Correctness	.54	.18	3.04	.002	.19, .89
Intercept	-1.29	.24	-5.53	<.001	-1.75, -.83
Change 3: Confidence					
GQS-Intelligence	.13	.03	4.02	<.001	.07, .20
Robot Feedback	-1.05	.08	-14.14	<.001	-1.20, -.91
GQS-Intelligence*Feedback	-.14	.05	-3.01	.003	-.23, -.05
Embodiment	.07	.07	1.12	.265	-.06, .20
Feedback*Embodiment	-.03	.09	-.35	.726	-.21, .15
Reversed Threat-ID	-.71	.29	-2.44	.015	-1.28, -.14
Feedback*Reversed Threat-ID	1.12	.30	3.78	<.001	.54, 1.71
Initial Threat-ID	.01	.05	.12	.904	-.08, .10
Initial Correctness	.12	.05	2.33	.020	.02, .22
Intercept	.35	.05	6.73	<.001	.25, .45

Note. $N = 135$. Multilevel models with all predictors and outcomes entered at Level 1, save for the between-subjects robot Embodiment variable at Level 2. All linear variables were standardized. Random intercept included to account for shared variance within participants; covariance matrices were unstructured. *Robot Feedback*: 0 = Agree, 1 = Disagree. *Embodiment*: 0 = Disembodied, 1 = Embodied. *Initial Threat-ID*: 0 = Ally, 1 = Enemy. *Initial Correctness*: 0 = Correct, 1 = Incorrect. *Reversed Threat-ID*: 0 = Repeated, 1 = Reversed. **Characterizing interactions: Participants who perceived the robot as intelligent were more confident when the robot agreed, and more likely to reverse their threat-ID and use of force decisions when the robot disagreed.**

Table S4

Parameter Estimates for Models Testing Interactions Between GQS-Anthropomorphism Appraisals and Robot Feedback on Changes in Threat-ID, Decisions to Kill, or Confidence (Expt. 1)

	Parm. Est.	*SE*	*t*	*p*	*95% CI*
Change 1: Threat-Identification					
GQS-Anthro	-.49	.13	-3.66	<.001	-.75, -.23
Robot Feedback	5.46	.53	10.33	<.001	4.42, 6.50
GQS-Anthro*Feedback	1.17	.36	3.21	.001	.45, 1.88
Embodiment	.09	.26	.33	.741	-.43, .60
Feedback*Embodiment	-.05	.68	-.08	.937	-1.39, 1.28
Initial Threat-ID	.46	.17	2.79	.005	.14, .79
Initial Correctness	.87	.19	4.74	<.001	.51, 1.24
Intercept	-1.30	.25	-5.28	<.001	-1.78, -.82
Change 2: Decisions to Kill					
GQS-Anthro	-.40	.13	-3.17	.002	-.65, -.15
Robot Feedback	5.00	.42	11.78	<.001	4.17, 5.83
GQS-Anthro*Feedback	.83	.30	2.78	.005	.24, 1.41
Embodiment	.05	.25	.19	.849	-.44 .54
Feedback*Embodiment	.10	.58	.17	.868	-1.05, 1.24
Initial Threat-ID	.73	.16	4.46	<.001	.41, 1.05
Initial Correctness	.57	.18	3.18	.002	.22, .92
Intercept	-1.35	.24	-5.72	<.001	-1.81, -.89
Change 3: Confidence					
GQS-Anthro	.04	.03	1.15	.250	-.03, .10
Robot Feedback	-1.01	.07	-13.65	<.001	-1.16, -.87
GQS-Anthro*Feedback	.02	.05	.45	.652	-.07, .11
Embodiment	.11	.06	1.70	.090	-.02, .24
Feedback*Embodiment	-.09	.09	-.94	.348	-.26, .09
Reversed Threat-ID	-.77	.29	-2.64	.008	-1.34, -.20
Feedback*Reversed Threat-ID	1.16	.30	3.88	<.001	.57, 1.74
Initial Threat-ID	.01	.05	.11	.913	-.08, .09
Initial Correctness	.12	.05	2.39	.017	.02, .22
Intercept	.33	.05	6.74	<.001	.23, .43

Note. *N* = 135. Multilevel models with all predictors and outcomes entered at Level 1, save for the between-subjects robot Embodiment variable at Level 2. All linear variables were standardized. Random intercept included to account for shared variance within participants; covariance matrices were unstructured. *Robot Feedback*: 0 = Agree, 1 = Disagree. *Embodiment*: 0 = Disembodied, 1 = Embodied. *Initial Threat-ID*: 0 = Ally, 1 = Enemy. *Initial Correctness*: 0 = Correct, 1 = Incorrect. *Reversed Threat-ID*: 0 = Repeated, 1 = Reversed. **Characterizing interactions: Participants who perceived the robot as anthropomorphic were more likely to reverse their threat-ID and use of force decisions when the robot disagreed.**

Table S5

Parameter Estimates for Models Testing Interactions Between GQS-Animacy Appraisals and Robot Feedback on Changes in Threat-ID, Decisions to Kill, or Confidence (Expt. 1)

	Parm. Est.	*SE*	*t*	*p*	*95% CI*
Change 1: Threat-Identification					
GQS-Animacy	-.38	.14	-2.83	.005	-.65, -.12
Robot Feedback	5.23	.48	10.94	<.001	4.29, 6.17
GQS-Animacy*Feedback	.42	.30	1.39	.165	-.17, 1.01
Embodiment	.08	.27	.28	.777	-.45 .60
Feedback*Embodiment	-.03	.67	-.05	.962	-1.35, 1.29
Initial Threat-ID	.47	.17	2.82	.005	.14, .79
Initial Correctness	.85	.18	4.65	<.001	.49, 1.21
Intercept	-1.27	.25	-5.18	<.001	-1.75, -.79
Change 2: Decisions to Kill					
GQS-Animacy	-.28	.13	-2.23	.026	-.53, -.03
Robot Feedback	4.91	.41	12.05	<.001	4.11, 5.71
GQS-Animacy*Feedback	.34	.27	1.29	.198	-.18, .87
Embodiment	.05	.25	.18	.857	-.45 .54
Feedback*Embodiment	.08	.58	.14	.892	-1.06, 1.21
Initial Threat-ID	.73	.16	4.50	<.001	.41, 1.05
Initial Correctness	.55	.18	3.09	.002	.20, .90
Intercept	-1.33	.24	-5.62	<.001	-1.79, -.87
Change 3: Confidence					
GQS-Animacy	.04	.03	1.35	.179	-.02, .11
Robot Feedback	-1.02	.07	-13.74	<.001	-1.17, -.88
GQS-Animacy*Feedback	.01	.05	-.10	.918	-.10, .09
Embodiment	.10	.07	1.58	.114	-.03, .23
Feedback*Embodiment	-.08	.09	-.86	.388	-.26, .10
Reversed Threat-ID	-.79	.29	-2.72	.007	-1.36, -.22
Feedback*Reversed Threat-ID	1.19	.30	4.00	<.001	.61, 1.77
Initial Threat-ID	.01	.05	.11	.911	-.08, .10
Initial Correctness	.12	.05	2.32	.021	.02, .22
Intercept	.33	.05	6.43	<.001	.23, .43

Note. *N* = 135. Multilevel models with all predictors and outcomes entered at Level 1, save for the between-subjects robot Embodiment variable at Level 2. All linear variables were standardized. Random intercept included to account for shared variance within participants; covariance matrices were unstructured. *Robot Feedback*: 0 = Agree, 1 = Disagree. *Embodiment*: 0 = Disembodied, 1 = Embodied. *Initial Threat-ID*: 0 = Ally, 1 = Enemy. *Initial Correctness*: 0 = Correct, 1 = Incorrect. *Reversed Threat-ID*: 0 = Repeated, 1 = Reversed.

Table S6

Parameter Estimates for Models Testing Interactions Between GQS-Likeability Appraisals and Robot Feedback on Changes in Threat-ID, Decisions to Kill, or Confidence (Expt. 1)

	Parm. Est.	*SE*	*t*	*p*	*95% CI*
Change 1: Threat-Identification					
GQS-Likeability	-.16	.13	-1.20	.232	-.42, .10
Robot Feedback	5.47	.52	10.57	<.001	4.46, 6.49
GQS-Likeability*Feedback	.57	.28	2.03	.043	.02, 1.13
Embodiment	.20	.27	.75	.451	-.32 .72
Feedback*Embodiment	-.25	.69	-.36	.718	-1.59, 1.10
Initial Threat-ID	.47	.17	2.86	.004	.15, .80
Initial Correctness	.83	.18	4.55	<.001	.47, 1.19
Intercept	-1.32	.25	-5.32	<.001	-1.81, -.83
Change 2: Decisions to Kill					
GQS-Likeability	-.19	.13	-1.54	.125	-.44, .05
Robot Feedback	5.04	.42	11.88	<.001	4.21, 5.87
GQS-Likeability*Feedback	.48	.25	1.91	.056	-.01, .96
Embodiment	.14	.25	.54	.589	-.36 .63
Feedback*Embodiment	-.05	.58	-.09	.931	-1.19, 1.09
Initial Threat-ID	.74	.16	4.51	<.001	.42, 1.06
Initial Correctness	.54	.18	3.01	.003	.19, .88
Intercept	-1.37	.24	-5.75	<.001	-1.83, -.90
Change 3: Confidence					
GQS-Likeability	.06	.03	1.93	.054	-.00, .13
Robot Feedback	-1.03	.07	-14.01	<.001	-1.17, -.88
GQS-Likeability*Feedback	-.04	.05	-.93	.351	-.13, .05
Embodiment	.11	.06	1.77	.077	-.01, .24
Feedback*Embodiment	-.08	.09	-.86	.392	-.26, .10
Reversed Threat-ID	-.76	.29	-2.61	.009	-1.33, -.19
Feedback*Reversed Threat-ID	1.17	.30	3.92	<.001	.58, 1.75
Initial Threat-ID	.01	.05	.17	.864	-.08, .10
Initial Correctness	.12	.05	2.35	.019	.02, .22
Intercept	.33	.05	6.34	<.001	.23, .43

Note. *N* = 135. Multilevel models with all predictors and outcomes entered at Level 1, save for the between-subjects robot Embodiment variable at Level 2. All linear variables were standardized. Random intercept included to account for shared variance within participants; covariance matrices were unstructured. *Robot Feedback*: 0 = Agree, 1 = Disagree. *Embodiment*: 0 = Disembodied, 1 = Embodied. *Initial Threat-ID*: 0 = Ally, 1 = Enemy. *Initial Correctness*: 0 = Correct, 1 = Incorrect. *Reversed Threat-ID*: 0 = Repeated, 1 = Reversed. **Characterizing interactions: Participants who perceived the robot as likable were modestly more likely to reverse their threat-identification decisions when the robot disagreed.**

Table S7

Parameter Estimates for Models Testing Interactions Between GQS-Safety Appraisals and Robot Feedback on Changes in Threat-ID, Decisions to Kill, or Confidence (Expt. 1)

	Parm. Est.	*SE*	*t*	*p*	*95% CI*
Change 1: Threat-Identification					
GQS-Safety	-.36	.13	-2.73	.006	-.62, -.10
Robot Feedback	5.46	.51	10.61	<.001	4.45, 6.47
GQS-Safety*Feedback	.83	.28	2.95	.003	.28, 1.39
Embodiment	.18	.27	.69	.490	-.34, .70
Feedback*Embodiment	-.18	.68	-.26	.794	-1.51, 1.16
Initial Threat-ID	.50	.17	3.02	.003	.18, .83
Initial Correctness	.83	.18	4.56	<.001	.48, 1.19
Intercept	-1.34	.25	-5.39	<.001	-1.82, -.85
Change 2: Decisions to Kill					
GQS-Safety	-.34	.13	-2.68	.007	-.58, -.09
Robot Feedback	5.06	.43	11.83	<.001	4.22, 5.90
GQS-Safety*Feedback	.69	.24	2.84	.005	.21, 1.16
Embodiment	.12	.25	.50	.620	-.37, .62
Feedback*Embodiment	-.02	.58	-.03	.975	-1.16, 1.12
Initial Threat-ID	.76	.16	4.66	<.001	.44, 1.09
Initial Correctness	.54	.18	3.01	.003	.19, .89
Intercept	-1.38	.24	-5.82	<.001	-1.85, -.92
Change 3: Confidence					
GQS-Safety	.06	.03	1.84	.065	-.01, .12
Robot Feedback	-1.02	.07	-13.87	<.001	-1.16, -.88
GQS-Safety*Feedback	-.01	.05	-.14	.886	-.10, .08
Embodiment	.12	.06	1.79	.074	-.01, .24
Feedback*Embodiment	-.08	.09	-.89	.374	-.26, .10
Reversed Threat-ID	-.76	.29	-2.60	.009	-1.33, -.19
Feedback*Reversed Threat-ID	1.15	.30	3.87	<.001	.57, 1.74
Initial Threat-ID	.02	.05	.29	.771	-.08, .10
Initial Correctness	.12	.05	2.36	.018	.02, .22
Intercept	.32	.05	6.27	<.001	.22, .42

Note. *N* = 135. Multilevel models with all predictors and outcomes entered at Level 1, save for the between-subjects robot Embodiment variable at Level 2. All linear variables were standardized. Random intercept included to account for shared variance within participants; covariance matrices were unstructured. *Robot Feedback*: 0 = Agree, 1 = Disagree. *Embodiment*: 0 = Disembodied, 1 = Embodied. *Initial Threat-ID*: 0 = Ally, 1 = Enemy. *Initial Correctness*: 0 = Correct, 1 = Incorrect. *Reversed Threat-ID*: 0 = Repeated, 1 = Reversed. (We omitted one GQS item from the Safety scale that used the contrastive anchors “Quiescent / Surprised” due to concern with its face validity.) **Characterizing interactions: Participants who perceived the robot as safe were more likely to reverse their threat-ID and use of force decisions when the robot disagreed.**

Table S8

Parameter Estimates for Models Testing Interactions Between PAS-High Expectations and Robot Feedback on Changes in Threat-ID, Decisions to Kill, or Confidence (Expt. 1)

	Parm. Est.	*SE*	*t*	*p*	*95% CI*
Change 1: Threat-Identification					
PAS-HE	-.40	.14	-2.97	.003	-.67, -.14
Robot Feedback	5.29	.48	10.97	<.001	4.34, 6.24
PAS-HE*Feedback	.21	.30	.69	.491	-.39, .80
Embodiment	.24	.26	.93	.351	-.27, .75
Feedback*Embodiment	-.14	.67	-.21	.831	-1.47, 1.18
Initial Threat-ID	.47	.17	2.87	.004	.15, .80
Initial Correctness	.83	.18	4.54	<.001	.47, 1.19
Intercept	-1.34	.25	-5.49	<.001	-1.82, -.86
Change 2: Decisions to Kill					
PAS-HE	-.25	.13	-1.97	.050	-.50, .00
Robot Feedback	4.97	.41	12.09	<.001	4.16, 5.77
PAS-HE*Feedback	-.00	.26	-.01	.991	-.52, .51
Embodiment	.16	.25	.66	.509	-.32, .65
Feedback*Embodiment	.01	.58	.01	.989	-1.13, 1.14
Initial Threat-ID	.74	.16	4.54	<.001	.42, 1.06
Initial Correctness	.53	.18	2.98	.003	.18, .88
Intercept	-1.38	.24	-5.84	<.001	-1.84, -.91
Change 3: Confidence					
PAS-HE	.07	.03	2.06	.039	.00, .13
Robot Feedback	-1.01	.07	-13.80	<.001	-1.15, -.87
PAS-HE*Feedback	.03	.05	.73	.464	-.06, .12
Embodiment	.12	.06	1.91	.057	-.00, .25
Feedback*Embodiment	-.08	.09	-.88	.382	-.26, .10
Reversed Threat-ID	-.81	.29	-2.79	.005	-1.37, -.24
Feedback*Reversed Threat-ID	1.19	.30	4.00	<.001	.61, 1.77
Initial Threat-ID	.00	.05	.01	.989	-.09, .09
Initial Correctness	.12	.05	2.27	.023	.02, .22
Intercept	.33	.05	6.37	<.001	.23, .43

Note. *N* = 135. Multilevel models with all predictors and outcomes entered at Level 1, save for the between-subjects robot Embodiment variable at Level 2. All linear variables were standardized. Random intercept included to account for shared variance within participants; covariance matrices were unstructured. *Robot Feedback*: 0 = Agree, 1 = Disagree. *Embodiment*: 0 = Disembodied, 1 = Embodied. *Initial Threat-ID*: 0 = Ally, 1 = Enemy. *Initial Correctness*: 0 = Correct, 1 = Incorrect. *Reversed Threat-ID*: 0 = Repeated, 1 = Reversed. The Perfect Automation Schema-High Expectations (*PAS-HE*; Merritt et al., 2015) subscale measures individual differences in expectations of the successful performance of automated systems, e.g., "Automated systems rarely make mistakes" (1=Strongly disagree; 2 =Disagree; 3=Somewhat disagree; 4=Neither agree nor disagree; 5=Somewhat agree; 6=Agree; 7=Strongly agree; four items, α = .74).

Table S9

Parameter Estimates for Models Testing Interactions Between PAS-All-or-None Appraisals and Robot Feedback on Changes in Threat-ID, Decisions to Kill, or Confidence (Expt. 1)

	Parm. Est.	*SE*	*t*	*p*	*95% CI*
Change 1: Threat-Identification					
PAS-AN	-.16	.13	-1.19	.235	-.42, .10
Robot Feedback	5.97	.65	9.19	<.001	4.70, 7.25
PAS-AN*Feedback	-.82	.34	-2.42	.016	-1.49, -.16
Embodiment	.19	.26	.70	.486	-.33, .70
Feedback*Embodiment	-.55	.73	-.75	.454	-1.97, .88
Initial Threat-ID	.47	.17	2.86	.004	.15, .80
Initial Correctness	.81	.18	4.46	<.001	.46, 1.17
Intercept	-1.29	.25	-5.24	<.001	-1.78, -.81
Change 2: Decisions to Kill					
PAS-AN	-.04	.13	-.29	.775	-.28, .21
Robot Feedback	5.25	.46	11.32	<.001	4.34, 6.16
PAS-AN*Feedback	-.64	.28	-2.29	.022	-1.18, -.09
Embodiment	.15	.25	.59	.557	-.35, .65
Feedback*Embodiment	-.16	.59	-.27	.788	-1.32, 1.00
Initial Threat-ID	.74	.16	4.56	<.001	.42, 1.06
Initial Correctness	.53	.18	2.96	.003	.18, .88
Intercept	-1.37	.24	-5.71	<.001	-1.84, -.90
Change 3: Confidence					
PAS-AN	.07	.03	2.22	.026	.01, .14
Robot Feedback	-1.01	.07	-13.84	<.001	-1.16, -.87
PAS-AN*Feedback	.04	.05	.92	.357	-.05, .13
Embodiment	.11	.06	1.66	.098	-.02, .23
Feedback*Embodiment	-.09	.09	-.99	.324	-.27, .09
Reversed Threat-ID	-.87	.29	-2.98	.003	-1.44, -.30
Feedback*Reversed Threat-ID	1.26	.30	4.23	<.001	.68, 1.84
Initial Threat-ID	-.00	.05	-.05	.962	-.09, .09
Initial Correctness	.11	.05	2.09	.037	.01, .21
Intercept	.34	.05	6.59	<.001	.24, .44

Note. *N* = 135. Multilevel models with all predictors and outcomes entered at Level 1, save for the between-subjects robot Embodiment variable at Level 2. All linear variables were standardized. Random intercept included to account for shared variance within participants; covariance matrices were unstructured. *Robot Feedback*: 0 = Agree, 1 = Disagree. *Embodiment*: 0 = Disembodied, 1 = Embodied. *Initial Threat-ID*: 0 = Ally, 1 = Enemy. *Initial Correctness*: 0 = Correct, 1 = Incorrect. *Reversed Threat-ID*: 0 = Repeated, 1 = Reversed. The Perfect Automation Schema-All-or-none (*PAS-AN*; Merritt et al., 2015) subscale measures individual differences in attitudes that automated systems either work completely or not at all, e.g., "If an automated system makes an error, then it is broken" (1=Strongly disagree; 2 =Disagree; 3=Somewhat disagree; 4=Neither agree nor disagree; 5=Somewhat agree; 6=Agree; 7=Strongly agree; four items, α = .65). **Characterizing interactions: Participants higher in PAS-AN ratings were modestly more likely to reverse their threat-identification or use of force decisions when the robot agreed.**

Table S10

Parameter Estimates for Models Testing Interactions Between Sex and Robot Feedback on Changes in Threat-ID, Decisions to Kill, or Confidence (Expt. 1)

	Parm. Est.	*SE*	*t*	*p*	*95% CI*
Change 1: Threat-Identification					
Sex	.60	.32	1.89	.059	-.02, 1.22
Robot Feedback	5.35	.50	10.63	<.001	4.36, 6.33
Sex*Feedback	-.44	.85	-.51	.607	-2.10, 1.23
Embodiment	.15	.26	.58	.561	-.36, .67
Feedback*Embodiment	-.08	.67	-.12	.902	-1.40, 1.24
Initial Threat-ID	.48	.17	2.92	.004	.16, .81
Initial Correctness	.83	.18	4.54	<.001	.47, 1.19
Intercept	-1.42	.25	-5.68	<.001	-1.92, -.93
Change 2: Decisions to Kill					
Sex	.49	.30	1.62	.105	-.10, 1.08
Robot Feedback	4.95	.42	11.78	<.001	4.12, 5.77
Sex*Feedback	.04	.81	.05	.962	-1.55, 1.63
Embodiment	.10	.25	.42	.678	-.39, .59
Feedback*Embodiment	.01	.58	.02	.985	-1.12, 1.15
Initial Threat-ID	.75	.16	4.58	<.001	.43, 1.06
Initial Correctness	.53	.18	3.00	.003	.19, .88
Intercept	-1.45	.24	-6.03	<.001	-1.93, -.98
Change 3: Confidence					
Sex	.22	.08	2.82	.005	.07, .37
Robot Feedback	-.98	.20	-4.81	<.001	-1.38, -.58
Sex*Feedback	-.02	.11	-.20	.843	-.24, .20
Embodiment	.10	.06	1.53	.126	-.03, .22
Feedback*Embodiment	-.08	.09	-.87	.382	-.26, .10
Reversed Threat-ID	-.79	.29	-2.75	.006	-1.36, -.23
Feedback*Reversed Threat-ID	1.19	.30	4.01	<.001	.61, 1.77
Initial Threat-ID	.00	.05	.08	.937	-.09, .09
Initial Correctness	.11	.05	2.18	.029	.01, .21
Intercept	-.06	.15	-.39	.697	-.34, .23

Note. *N* = 135. Multilevel models with all predictors and outcomes entered at Level 1, save for the between-subjects robot Embodiment variable at Level 2. All linear variables were standardized. Random intercept included to account for shared variance within participants; covariance matrices were unstructured. *Robot Feedback*: 0 = Agree, 1 = Disagree. *Embodiment*: 0 = Disembodied, 1 = Embodied. *Initial Threat-ID*: 0 = Ally, 1 = Enemy. *Initial Correctness*: 0 = Correct, 1 = Incorrect. *Reversed Threat-ID*: 0 = Repeated, 1 = Reversed. *Sex*: 1 = Male, 2 = Female. **Sex did not predict reversing either threat-identification or use of force decisions. Women showed a modest overall increase in confidence following robot feedback, although there was no interaction between sex and robot feedback on shifts in confidence.**

Table S11

Parameter Estimates for Models Testing Interactions Between Drone Warfare Attitudes and Robot Feedback on Changes in Threat-ID, Decisions to Kill, or Confidence (Expt. 1)

	Parm. Est.	*SE*	*t*	*p*	*95% CI*
Change 1: Threat-Identification					
Drone Attitudes	.40	.13	2.99	.003	.14, .66
Robot Feedback	5.67	.55	10.33	<.001	4.59, 6.75
Drone Attitudes*Feedback	-1.28	.38	-3.36	<.001	-2.03, -.53
Embodiment	.24	.27	.91	.365	-.28, .76
Feedback*Embodiment	-.23	.69	-.34	.732	-1.58, 1.11
Initial Threat-ID	.50	.17	2.99	.003	.17, .83
Initial Correctness	.89	.19	4.78	<.001	.52, 1.25
Intercept	-1.40	.25	-5.60	<.001	-1.89, -.91
Change 2: Decisions to Kill					
Drone Attitudes	.30	.13	2.40	.016	.06, .55
Robot Feedback	5.05	.42	11.96	<.001	4.23, 5.88
Drone Attitudes*Feedback	-.63	.29	-2.14	.033	-1.20, -.05
Embodiment	.17	.25	.67	.503	-.32, .66
Feedback*Embodiment	-.09	.58	-.16	.874	-1.23, 1.05
Initial Threat-ID	.76	.16	4.64	<.001	.44, 1.08
Initial Correctness	.57	.18	3.16	.002	.22, .92
Intercept	-1.42	.24	-5.95	<.001	-1.89, -.95
Change 3: Confidence					
Drone Attitudes	-.04	.03	-1.25	.213	-.10, .02
Robot Feedback	-1.03	.07	-13.96	<.001	-1.17, -.88
Drone Attitudes*Feedback	.04	.05	.79	.433	-.05, .13
Embodiment	.12	.06	1.90	.057	-.00, .25
Feedback*Embodiment	-.09	.09	-.93	.351	-.26, .09
Reversed Threat-ID	-.76	.29	-2.62	.009	-1.33, -.19
Feedback*Reversed Threat-ID	1.17	.30	3.93	<.001	.59, 1.76
Initial Threat-ID	.01	.05	.23	.819	-.08, .10
Initial Correctness	.12	.05	2.26	.024	.02, .22
Intercept	.32	.05	6.24	<.001	.22, .42

Note. $N = 135$. Multilevel models with all predictors and outcomes entered at Level 1, save for the between-subjects robot Embodiment variable at Level 2. All linear variables were standardized. Random intercept included to account for shared variance within participants; covariance matrices were unstructured. *Robot Feedback*: 0 = Agree, 1 = Disagree. *Embodiment*: 0 = Disembodied, 1 = Embodied. *Initial Threat-ID*: 0 = Ally, 1 = Enemy. *Initial Correctness*: 0 = Correct, 1 = Incorrect. *Reversed Threat-ID*: 0 = Repeated, 1 = Reversed. **Characterizing interactions: Participants who disapproved of drone warfare were less likely to reverse their threat-ID or use of force decisions when the robot disagreed.**

Table S12

Parameter Estimates for Multilevel Models Testing Interactions Between GQS-Intelligence and Robot Feedback on Changes in Threat-ID, Decisions to Kill, or Confidence (Expt. 2)

	Parm. Est.	*SE*	*t*	*p*	*95% CI*
Change 1: Threat-Identification					
GQS-Intelligence	-.61	.07	-8.76	<.001	-.74, -.47
Robot Feedback	6.52	.49	13.23	<.001	5.55, 7.48
GQS-Intelligence*Feedback	.39	.16	2.37	.018	.07, .71
Interactive Humanoid	.15	.16	.91	.363	-.17, .47
Interactive Nonhumanoid	.04	.17	.21	.831	-.29, .36
Feedback*Interact. Human.	-.88	.35	-2.49	.013	-1.57, -.19
Feedback*Interact. Nonhum.	-1.20	.40	-2.99	.003	-1.99, -.41
Initial Threat-ID	.44	.09	4.75	<.001	.26, .63
Initial Correctness	.72	.10	7.11	<.001	.52, .92
Intercept	-1.68	.22	-7.59	<.001	-2.11, -1.24
Change 2: Decisions to Kill					
GQS-Intelligence	-.58	.06	-9.20	<.001	-.71, -.46
Robot Feedback	5.44	.43	12.69	<.001	4.60, 6.28
GQS-Intelligence*Feedback	.71	.14	4.96	<.001	.43, .98
Interactive Humanoid	.30	.15	2.02	.043	.01, .60
Interactive Nonhumanoid	.17	.15	1.09	.278	-.13, .46
Feedback* Interact. Human.	-.67	.35	-1.91	.057	-1.36, .02
Feedback* Interact. Nonhum.	-.21	.34	-.61	.541	-.86, .45
Initial Threat-ID	.86	.09	9.33	<.001	.68, 1.04
Initial Correctness	.68	.10	6.96	<.001	.49, .88
Intercept	-2.00	.21	-9.68	<.001	-2.40, -1.59
Change 3: Confidence					
GQS-Intelligence	.10	.02	5.60	<.001	.06, .13
Robot Feedback	-.95	.05	-19.51	<.001	-1.04, -.85
GQS-Intelligence*Feedback	-.07	.02	-2.67	.008	-.11, -.02
Interactive Humanoid	-.03	.04	-.64	.525	-.11, .05
Interactive Nonhumanoid	-.10	.04	-2.44	.015	-.18, -.02
Feedback* Interact. Human.	-.13	.06	-2.30	.021	-.25, -.02
Feedback* Interact. Nonhum.	.02	.06	-.30	.761	-.13, .10
Reversed Threat-ID	-.72	.12	-6.21	<.001	-.94, -.49
Feedback*Reversed Threat-ID	1.21	.12	9.99	<.001	.97, 1.45
Initial Threat-ID	-.08	.02	-3.20	.001	-.12, -.03
Initial Correctness	.10	.03	3.81	<.001	.05, .15
Intercept	.39	.03	11.61	<.001	.32, .46

Note. *N* = 423. Multilevel models with predictors and outcomes entered at Level 1, except between-subjects variables (Interactive Humanoid, Interactive Nonhumanoid) at Level 2. The Interactive Humanoid and Interactive Nonhumanoid conditions were dummy-coded with Nonhumanoid as the control category. All linear variables were standardized. Random intercept included to account for the shared variance within participants; covariance matrices were unstructured. *Robot Feedback*: 0 = Agree, 1 = Disagree. *Initial Threat-ID*: 0 = Ally, 1 = Enemy. *Initial Correctness*: 0 = Correct, 1 = Incorrect. *Reversed Threat-ID*: 0 = Repeated, 1 = Reversed. **Characterizing interactions: Participants who perceived the robot as intelligent were more confident when the robot agreed, and more likely to reverse their threat-ID and use of force decisions when the robot disagreed.**

Table S13

Parameter Estimates for Multilevel Models Testing Interactions Between GQS-Anthropomorphism and Robot Feedback on Changes in Threat-ID, Decisions to Kill, or Confidence (Expt. 2)

	Parm. Est.	*SE*	*t*	*p*	*95% CI*
Change 1: Threat-Identification					
GQS-Anthro	-.17	.07	-2.30	.021	-.31, -.03
Robot Feedback	6.93	.51	13.51	<.001	5.92, 7.93
GQS-Anthro*Feedback	-.26	.14	-1.79	.073	-.54, .02
Interactive Humanoid	.12	.18	.71	.476	-.22, .47
Interactive Nonhumanoid	.17	.18	.96	.339	-.18, .51
Feedback*Interact. Human.	-1.05	.37	-2.87	.004	-1.77, -.33
Feedback*Interact. Nonhum.	-1.43	.41	-3.52	<.001	-2.22, -.63
Initial Threat-ID	.44	.09	4.76	<.001	.26, .63
Initial Correctness	.73	.10	7.22	<.001	.53, .93
Intercept	-1.74	.23	-7.49	<.001	-2.20, -1.29
Change 2: Decisions to Kill					
GQS-Anthro	-.24	.07	-3.64	<.001	-.37, -.11
Robot Feedback	5.77	.45	12.91	<.001	4.90, 6.65
GQS-Anthro*Feedback	-.13	.14	-.94	.350	-.39, .14
Interactive Humanoid	.25	.16	1.58	.115	-.06, .55
Interactive Nonhumanoid	.27	.16	1.71	.088	-.04, .58
Feedback* Interact. Human.	-.77	.36	-2.12	.034	-1.47, -.06
Feedback* Interact. Nonhum.	-.48	.34	-1.41	.159	-1.14, .19
Initial Threat-ID	.84	.09	9.25	<.001	.67, 1.02
Initial Correctness	.69	.10	7.05	<.001	.50, .88
Intercept	-2.00	.22	-9.42	<.001	-2.42, -1.59
Change 3: Confidence					
GQS-Anthro	-.05	.02	-2.99	.003	-.08, -.02
Robot Feedback	-.93	.05	-19.33	<.001	-1.03, -.84
GQS-Anthro*Feedback	.07	.02	3.00	.003	.03, .12
Interactive Humanoid	-.01	.04	-.14	.888	-.09, .08
Interactive Nonhumanoid	-.06	.04	-1.49	.137	-.14, .02
Feedback* Interact. Human.	-.16	.06	-2.71	.007	-.27, -.04
Feedback* Interact. Nonhum.	-.05	.06	-.92	.357	-.17, .06
Reversed Threat-ID	-.68	.12	-5.87	<.001	-.91, -.45
Feedback*Reversed Threat-ID	1.19	.12	9.77	<.001	.95, 1.42
Initial Threat-ID	-.07	.03	-3.05	.002	-.12, -.03
Initial Correctness	.11	.03	4.16	<.001	.06, .15
Intercept	.36	.03	10.77	<.001	.30, .43

Note. $N = 423$. Multilevel models with predictors and outcomes entered at Level 1, except between-subjects variables (Interactive Humanoid, Interactive Nonhumanoid) at Level 2. The Interactive Humanoid and Interactive Nonhumanoid conditions were dummy-coded with Nonhumanoid as the control category. All linear variables were standardized. Random intercept included to account for the shared variance within participants; covariance matrices were unstructured. *Robot Feedback*: 0 = Agree, 1 = Disagree. *Initial Threat-ID*: 0 = Ally, 1 = Enemy. *Initial Correctness*: 0 = Correct, 1 = Incorrect. *Reversed Threat-ID*: 0 = Repeated, 1 = Reversed. **Characterizing interaction: Participants who perceived the robot as anthropomorphic were more confident when the robot agreed.**

Table S14

Parameter Estimates for Multilevel Models Testing Interactions Between GQS-Animacy and Robot Feedback on Changes in Threat-ID, Decisions to Kill, or Confidence (Expt. 2)

	Parm. Est.	*SE*	*t*	*p*	*95% CI*
Change 1: Threat-Identification					
GQS-Animacy	-.27	.07	-3.81	<.001	-.42, -.13
Robot Feedback	6.95	.52	13.30	<.001	5.92, 7.97
GQS-Animacy*Feedback	-.22	.16	-1.39	.165	-.52, .09
Interactive Humanoid	.09	.17	.49	.625	-.25, .42
Interactive Nonhumanoid	.11	.18	.65	.515	-.23, .46
Feedback*Interact. Human.	-1.07	.37	-2.88	.004	-1.79, -.34
Feedback*Interact. Nonhum.	-1.44	.41	-3.52	<.001	-2.24, .64
Initial Threat-ID	.44	.09	4.72	<.001	.26, .62
Initial Correctness	.73	.10	7.23	<.001	.53, .93
Intercept	-1.68	.23	-7.26	<.001	-2.13, -1.23
Change 2: Decisions to Kill					
GQS-Animacy	-.30	.07	-4.64	<.001	-.43, -.17
Robot Feedback	5.72	.45	12.71	<.001	4.84, 6.61
GQS-Animacy*Feedback	-.04	.15	-.29	.772	-.33, .24
Interactive Humanoid	.21	.16	1.38	.169	-.09, .52
Interactive Nonhumanoid	.22	.16	1.39	.165	-.09, .53
Feedback* Interact. Human.	-.74	.36	-2.05	.041	-1.45, -.03
Feedback* Interact. Nonhum.	-.45	.34	-1.33	.184	-1.12, .22
Initial Threat-ID	.84	.09	9.21	<.001	.66, 1.02
Initial Correctness	.69	.10	7.05	<.001	.50, .88
Intercept	-1.95	.21	-9.18	<.001	-2.37, -1.53
Change 3: Confidence					
GQS-Animacy	-.00	.02	-.10	.921	-.04, .03
Robot Feedback	-.93	.05	-19.20	<.001	-1.03, -.84
GQS-Animacy*Feedback	.04	.02	1.72	.085	-.01, .09
Interactive Humanoid	-.02	.04	-.49	.623	-.10, .06
Interactive Nonhumanoid	-.07	.04	-1.75	.080	-.16, .01
Feedback* Interact. Human.	-.15	.06	-2.58	.010	-.27, -.04
Feedback* Interact. Nonhum.	-.05	.06	-.87	.385	-.17, .06
Reversed Threat-ID	-.70	.12	-6.04	<.001	-.93, -.47
Feedback*Reversed Threat-ID	1.20	.12	9.88	<.001	.96, 1.44
Initial Threat-ID	-.08	.02	-3.16	.002	-.12, -.03
Initial Correctness	.10	.03	4.03	<.001	.05, .15
Intercept	.38	.03	11.09	<.001	.31, .44

Note. N = 423. Multilevel models with predictors and outcomes entered at Level 1, except between-subjects variables (Interactive Humanoid, Interactive Nonhumanoid) at Level 2. The Interactive Humanoid and Interactive Nonhumanoid conditions were dummy-coded with Nonhumanoid as the control category. All linear variables were standardized. Random intercept included to account for the shared variance within participants; covariance matrices were unstructured. *Robot Feedback*: 0 = Agree, 1 = Disagree. *Initial Threat-ID*: 0 = Ally, 1 = Enemy. *Initial Correctness*: 0 = Correct, 1 = Incorrect. *Reversed Threat-ID*: 0 = Repeated, 1 = Reversed.

Table S15

Parameter Estimates for Multilevel Models Testing Interactions Between GQS-Likeability and Robot Feedback on Changes in Threat-ID, Decisions to Kill, or Confidence (Expt. 2)

	Parm. Est.	*SE*	*t*	*p*	*95% CI*
Change 1: Threat-Identification					
GQS-Likeability	-.35	.07	-4.98	<.001	-.49, -.21
Robot Feedback	6.56	.49	13.39	<.001	5.60, 7.52
GQS-Likeability*Feedback	.41	.16	2.57	.010	.10, .72
Interactive Humanoid	.18	.17	1.03	.302	-.16, .51
Interactive Nonhumanoid	.15	.17	.84	.403	-.20, .49
Feedback*Interact. Human.	-.88	.35	-2.47	.013	-1.57, -.18
Feedback*Interact. Nonhum.	-1.20	.40	-2.99	.003	-1.99, -.42
Initial Threat-ID	.43	.09	4.65	<.001	.25, .62
Initial Correctness	.74	.10	7.31	<.001	.54, .94
Intercept	-1.77	.23	-7.72	<.001	-2.22, -1.32
Change 2: Decisions to Kill					
GQS-Likeability	-.38	.07	-5.93	<.001	-.51, -.26
Robot Feedback	5.54	.43	12.89	<.001	4.70, 6.38
GQS-Likeability*Feedback	.56	.15	3.85	<.001	.28, .85
Interactive Humanoid	.33	.16	2.10	.036	.02, .63
Interactive Nonhumanoid	.26	.16	1.64	.100	-.05, .57
Feedback* Interact. Human.	-.69	.35	-1.94	.053	-1.38, .01
Feedback* Interact. Nonhum.	-.29	.34	-.86	.390	-.94, .37
Initial Threat-ID	.84	.09	9.13	<.001	.66, 1.02
Initial Correctness	.71	.10	7.19	<.001	.51, .90
Intercept	-2.07	.21	-9.75	<.001	-2.48, -1.65
Change 3: Confidence					
GQS-Likeability	.06	.02	3.60	<.001	.03, .09
Robot Feedback	-.95	.05	-19.66	<.001	-1.04, -.85
GQS-Likeability*Feedback	-.04	.02	-1.54	.125	-.08, .01
Interactive Humanoid	-.02	.04	-.51	.608	-.10, .06
Interactive Nonhumanoid	-.09	.04	-2.07	.038	-.17, -.01
Feedback* Interact. Human.	-.14	.06	-2.36	.018	-.25, -.02
Feedback* Interact. Nonhum.	-.03	.06	-.51	.612	-.15, .09
Reversed Threat-ID	-.70	.12	-6.02	<.001	-.92, -.47
Feedback*Reversed Threat-ID	1.20	.12	9.88	<.001	.96, 1.44
Initial Threat-ID	-.08	.02	-3.21	.001	-.12, -.03
Initial Correctness	.10	.03	4.04	<.001	.05, .15
Intercept	.38	.03	11.34	<.001	.32, .45

Note. *N* = 423. Multilevel models with predictors and outcomes entered at Level 1, except between-subjects variables (Interactive Humanoid, Interactive Nonhumanoid) at Level 2. The Interactive Humanoid and Interactive Nonhumanoid conditions were dummy-coded with Nonhumanoid as the control category. All linear variables were standardized. Random intercept included to account for the shared variance within participants; covariance matrices were unstructured. *Robot Feedback*: 0 = Agree, 1 = Disagree. *Initial Threat-ID*: 0 = Ally, 1 = Enemy. *Initial Correctness*: 0 = Correct, 1 = Incorrect. *Reversed Threat-ID*: 0 = Repeated, 1 = Reversed. **Characterizing interactions: Participants who perceived the robot as likable were more likely to reverse their threat-ID and use of force decisions when the robot disagreed.**

Table S16

Parameter Estimates for Multilevel Models Testing Interactions Between GQS-Safety and Robot Feedback on Changes in Threat-ID, Decisions to Kill, or Confidence (Expt. 2)

	Parm. Est.	*SE*	*t*	*p*	*95% CI*
Change 1: Threat-Identification					
GQS-Safety	-.30	.07	-4.18	<.001	-.44, -.16
Robot Feedback	6.89	.50	13.76	<.001	5.91, 7.87
GQS-Safety*Feedback	.57	.15	3.82	<.001	.28, .86
Interactive Humanoid	.27	.18	1.53	.127	-.08, .61
Interactive Nonhumanoid	.30	.18	1.72	.086	-.04, .65
Feedback*Interact. Human.	-1.09	.36	-3.01	.003	-1.80, -.38
Feedback*Interact. Nonhum.	-1.41	.40	-3.53	<.001	-2.20, -.63
Initial Threat-ID	.45	.09	4.79	<.001	.26 .63
Initial Correctness	.74	.10	7.31	<.001	.54, .94
Intercept	-1.94	.23	-8.30	<.001	-2.40, -1.48
Change 2: Decisions to Kill					
GQS-Safety	-.31	.06	-4.80	<.001	-.44, -.18
Robot Feedback	5.95	.45	13.32	<.001	5.07, 6.83
GQS-Safety*Feedback	.64	.14	4.70	<.001	.37, .90
Interactive Humanoid	.42	.16	2.63	.009	.11, .73
Interactive Nonhumanoid	.42	.16	2.62	.009	.11, .73
Feedback* Interact. Human.	-.95	.36	-2.60	.009	-1.66, -.23
Feedback* Interact. Nonhum.	-.57	.34	-1.68	.092	-1.23, .09
Initial Threat-ID	.85	.09	9.29	<.001	.67, 1.03
Initial Correctness	.70	.10	7.17	<.001	.51, .90
Intercept	-2.24	.22	-10.33	<.001	-2.66, -1.81
Change 3: Confidence					
GQS-Safety	.06	.02	3.46	<.001	.03, .09
Robot Feedback	-.94	.05	-19.48	<.001	-1.03, -.84
GQS-Safety*Feedback	-.08	.02	-3.28	.001	-.13, -.03
Interactive Humanoid	-.00	.04	-.05	.964	-.08, .08
Interactive Nonhumanoid	-.06	.04	-1.37	.171	-.14, .03
Feedback* Interact. Human.	-.16	.06	-2.78	.005	-.28, -.05
Feedback* Interact. Nonhum.	-.06	.06	-1.02	.307	-.18, .06
Reversed Threat-ID	-.68	.12	-5.90	<.001	-.91, -.46
Feedback*Reversed Threat-ID	1.20	.12	9.88	<.001	.96, 1.44
Initial Threat-ID	-.08	.02	3.16	.002	-.12, -.03
Initial Correctness	.10	.03	4.05	<.001	.05, .15
Intercept	.36	.03	10.78	<.001	.30, .43

Note. N = 423. Multilevel models with predictors and outcomes entered at Level 1, except between-subjects variables (Interactive Humanoid, Interactive Nonhumanoid) at Level 2. The Interactive Humanoid and Interactive Nonhumanoid conditions were dummy-coded with Nonhumanoid as the control category. All linear variables were standardized. Random intercept included to account for the shared variance within participants; covariance matrices were unstructured. *Robot Feedback*: 0 = Agree, 1 = Disagree. *Initial Threat-ID*: 0 = Ally, 1 = Enemy. *Initial Correctness*: 0 = Correct, 1 = Incorrect. *Reversed Threat-ID*: 0 = Repeated, 1 = Reversed. **Characterizing interactions: Participants who perceived the robot as safe were more confident when the robot agreed, and more likely to reverse their threat-ID and use of force decisions when the robot disagreed.**

Table S17

Parameter Estimates for Multilevel Models Testing Interactions Between Sex and Robot Feedback on Changes in Threat-ID, Decisions to Kill, or Confidence (Expt. 2)

	Parm. Est.	*SE*	*t*	*p*	*95% CI*
Change 1: Threat-Identification					
Sex	-.44	.14	-3.21	.001	-.70, -.17
Robot Feedback	6.06	.65	9.33	<.001	4.78, 7.33
Sex*Feedback	.43	.30	1.46	.146	-.15, 1.02
Interactive Humanoid	.20	.17	1.13	.261	-.15, .54
Interactive Nonhumanoid	.22	.18	1.27	.205	-.12, .57
Feedback*Interact. Human.	-.91	.36	2.57	.010	-1.61, -.22
Feedback*Interact. Nonhum.	-1.33	.40	-3.33	<.001	-2.11, -.55
Initial Threat-ID	.46	.09	4.88	<.001	.27, .64
Initial Correctness	.74	.10	7.30	<.001	.54, .94
Intercept	-1.21	.30	-4.08	<.001	-1.79, -.63
Change 2: Decisions to Kill					
Sex	-.28	.12	-2.28	.023	-.52, -.04
Robot Feedback	5.27	.59	8.99	<.001	4.12, 6.42
Sex*Feedback	.26	.27	.93	.350	-.28, .79
Interactive Humanoid	.33	.16	2.09	.037	.02, .64
Interactive Nonhumanoid	.33	.16	2.10	.036	.02, .65
Feedback* Interact. Human.	-.71	.35	-1.99	.047	-1.40, -.01
Feedback* Interact. Nonhum.	-.42	.33	-1.27	.204	-1.08, .23
Initial Threat-ID	.85	.09	9.34	<.001	.67, 1.03
Initial Correctness	.70	.10	7.14	<.001	.51, .89
Intercept	-1.70	.27	-6.27	<.001	-2.23, -1.17
Change 3: Confidence					
Sex	.13	.03	4.03	<.001	.07, .19
Robot Feedback	-.98	.08	-12.10	<.001	-1.14, -.82
Sex*Feedback	-.03	.05	.63	.531	-.06, .12
Interactive Humanoid	-.02	.04	-.36	.716	-.10, .07
Interactive Nonhumanoid	-.07	.04	-1.67	.095	-.15, .01
Feedback* Interact. Human.	-.14	.06	-2.34	.019	-.25, -.02
Feedback* Interact. Nonhum.	-.04	.06	-.61	.542	-.15, .08
Reversed Threat-ID	-.70	.12	-6.08	<.001	-.93, -.48
Feedback*Reversed Threat-ID	1.20	.12	9.90	<.001	.96, 1.43
Initial Threat-ID	-.07	.02	-3.13	.002	-.12, -.03
Initial Correctness	.10	.03	4.09	<.001	.05, .15
Intercept	.19	.06	3.21	.001	.07, .30

Note. *N* = 423. Multilevel models with predictors and outcomes entered at Level 1, except between-subjects variables (Interactive Humanoid, Interactive Nonhumanoid) at Level 2. The Interactive Humanoid and Interactive Nonhumanoid conditions were dummy-coded with Nonhumanoid as the control category. All linear variables were standardized. Random intercept included to account for the shared variance within participants; covariance matrices were unstructured. *Robot Feedback*: 0 = Agree, 1 = Disagree. *Initial Threat-ID*: 0 = Ally, 1 = Enemy. *Initial Correctness*: 0 = Correct, 1 = Incorrect. *Reversed Threat-ID*: 0 = Repeated, 1 = Reversed. *Sex*: 1 = Male, 2 = Female. **In small effects, women reversed their threat-identification and use of force decisions, and reported greater increases in confidence, relative to men. There was no interactions between sex and robot feedback.**

Table S18

Parameter Estimates for Multilevel Models Testing Interactions Between Drone Warfare Attitudes and Robot Feedback on Changes in Threat-ID, Decisions to Kill, or Confidence (Expt. 2)

	Parm. Est.	*SE*	*t*	*p*	*95% CI*
Change 1: Threat-Identification					
Drone Attitudes	.25	.07	3.50	<.001	.11, .39
Robot Feedback	6.65	.49	13.50	<.001	5.68, 7.61
Drone Attitudes*Feedback	-.11	.16	-.68	.494	-.41, .20
Interactive Humanoid	.13	.17	.76	.448	-.21, .47
Interactive Nonhumanoid	.17	.18	.99	.321	-.17, .52
Feedback*Interact. Human.	-.88	.36	-2.48	.013	-1.58, -.19
Feedback*Interact. Nonhum.	-1.30	.40	-3.26	.001	-2.09, -.52
Initial Threat-ID	.44	.09	4.76	<.001	.26, .63
Initial Correctness	.73	.10	7.26	<.001	.54, .93
Intercept	-1.76	.23	-7.60	<.001	-2.21, -1.30
Change 2: Decisions to Kill					
Drone Attitudes	.25	.06	3.97	<.001	.13, .38
Robot Feedback	5.59	.43	12.94	<.001	4.74, 6.43
Drone Attitudes*Feedback	-.20	.14	-1.41	.158	-.48, .08
Interactive Humanoid	.27	.16	1.74	.081	-.03, .58
Interactive Nonhumanoid	.29	.16	1.83	.067	-.02, .60
Feedback* Interact. Human.	-.66	.36	-1.87	.062	-1.36, .03
Feedback* Interact. Nonhum.	-.39	.33	-1.16	.246	-1.04, .27
Initial Threat-ID	.85	.09	9.25	<.001	.67, 1.02
Initial Correctness	.70	.10	7.10	<.001	.50, .89
Intercept	-2.04	.22	-9.58	<.001	-2.46, -1.62
Change 3: Confidence					
Drone Attitudes	-.03	.02	-1.46	.146	-.06, .01
Robot Feedback	-.94	.05	-19.54	<.001	-1.04, -.85
Drone Attitudes*Feedback	-.02	.02	-.87	.384	-.07, .03
Interactive Humanoid	-.03	.04	-.60	.550	-.11, .06
Interactive Nonhumanoid	-.08	.04	-1.86	.063	-.16, .00
Feedback* Interact. Human.	-.14	.06	-2.41	.016	-.25, -.03
Feedback* Interact. Nonhum.	-.04	.06	-.67	.501	-.16, .08
Reversed Threat-ID	-.70	.12	-6.08	<.001	-.93, -.48
Feedback*Reversed Threat-ID	1.20	.12	9.92	<.001	.97, 1.44
Initial Threat-ID	-.08	.02	-3.19	.001	-.12, -.03
Initial Correctness	.10	.03	4.03	<.001	.05, .15
Intercept	.38	.03	11.27	<.001	.31, .45

Note. *N* = 423. Multilevel models with predictors and outcomes entered at Level 1, except between-subjects variables (Interactive Humanoid, Interactive Nonhumanoid) at Level 2. The Interactive Humanoid and Interactive Nonhumanoid conditions were dummy-coded with Nonhumanoid as the control category. All linear variables were standardized. Random intercept included to account for the shared variance within participants; covariance matrices were unstructured. *Robot Feedback*: 0 = Agree, 1 = Disagree. *Initial Threat-ID*: 0 = Ally, 1 = Enemy. *Initial Correctness*: 0 = Correct, 1 = Incorrect. *Reversed Threat-ID*: 0 = Repeated, 1 = Reversed.

Table S19

Parameter Estimates for Multilevel Models Testing Interactions Between Appraisals of Personal Task-Competence and Robot Feedback on Changes in Threat-ID, Decisions to Kill, or Confidence (Expt. 2)

	Parm. Est.	*SE*	*t*	*p*	*95% CI*
Change 1: Threat-Identification					
Personal Task-competence	-.41	.07	-5.65	<.001	-.56, -.27
Robot Feedback	7.12	.51	13.90	<.001	6.11, 8.12
Personal Task-compet*Feedback	-.76	.16	-4.76	<.001	-1.07, -.45
Interactive Humanoid	.24	.17	1.38	.168	-.10, .58
Interactive Nonhumanoid	.34	.18	1.90	.057	-.01, .68
Feedback*Interact. Human.	-1.14	.36	-3.15	.002	-1.85, -.43
Feedback*Interact. Nonhum.	-1.65	.41	-4.04	<.001	-2.45, -.85
Initial Threat-ID	.46	.09	4.95	<.001	.28, .65
Initial Correctness	.75	.10	7.38	<.001	.55, .95
Intercept	-1.97	.23	-8.44	<.001	-2.42, -1.51
Change 2: Decisions to Kill					
Personal Task-competence	-.36	.07	-5.53	<.001	-.49, -.23
Robot Feedback	5.95	.44	13.39	<.001	5.08, 6.82
Personal Task-compet*Feedback	-.61	.15	-4.16	<.001	-.90. -.33
Interactive Humanoid	.39	.16	2.48	.013	.08, .69
Interactive Nonhumanoid	.45	.16	2.82	.005	.14, .76
Feedback* Interact. Human.	-.86	.36	-2.44	.015	-1.58, -.17
Feedback* Interact. Nonhum.	-.66	.34	-1.94	.053	-1.32, .01
Initial Threat-ID	.87	.09	9.46	<.001	.69, 1.05
Initial Correctness	.71	.10	7.21	<.001	.52, .90
Intercept	-2.24	.21	-10.49	<.001	-2.66, -1.82
Change 3: Confidence					
Personal Task-competence	.12	.02	7.17	<.001	.09, .15
Robot Feedback	-.92	.05	-19.30	<.001	-1.02, -.83
Personal Task-compet*Feedback	.06	.02	2.60	.009	.02, .11
Interactive Humanoid	.01	.04	.12	.908	-.08, .09
Interactive Nonhumanoid	-.03	.04	-.75	.455	-.11, .05
Feedback* Interact. Human.	-.15	.06	-2.60	.009	-.26, -.04
Feedback* Interact. Nonhum.	-.06	.06	-1.01	.314	-.18, .06
Reversed Threat-ID	-.66	.12	-5.75	<.001	-.89, -.44
Feedback*Reversed Threat-ID	1.15	.12	9.54	<.001	.91, 1.39
Initial Threat-ID	-.07	.02	-2.79	.005	-.11, -.02
Initial Correctness	.10	.03	4.10	<.001	.05, .15
Intercept	.35	.03	10.34	<.001	.28, .41

Note. N = 423. Multilevel models with predictors and outcomes entered at Level 1, except between-subjects variables (Interactive Humanoid, Interactive Nonhumanoid) at Level 2. The Interactive Humanoid and Interactive Nonhumanoid conditions were dummy-coded with Nonhumanoid as the control category. All linear variables were standardized. Random intercept included to account for the shared variance within participants; covariance matrices were unstructured. *Robot Feedback*: 0 = Agree, 1 = Disagree. *Initial Threat-ID*: 0 = Ally, 1 = Enemy. *Initial Correctness*: 0 = Correct, 1 = Incorrect. *Reversed Threat-ID*: 0 = Repeated, 1 = Reversed. *Item:* "In your view, which of the following best describes your personal ability to perform the visual task where you try to detect an enemy versus ally symbol?" (1=Perfect; 2=Good; 3=Fair; 4=Bad; 5=Terrible; composite scores were reversed; M = 2.85, SD = .89). **Characterizing interactions: Participants who perceived themselves as more task-competent were less likely to reverse their threat-ID and use of force decisions when the robot disagreed. They were also more confident when the robot disagreed and they did not reverse, less confident when the robot disagreed and they did reverse, and less confident when the robot agreed.**

Table S20

Parameter Estimates for Multilevel Models Testing Interactions Between Appraisals of Relative Task-Competence and Robot Feedback on Changes in Threat-ID, Decisions to Kill, or Confidence (Expt. 2)

	Parm. Est.	*SE*	*t*	*p*	*95% CI*
Change 1: Threat-Identification					
Relative Task-competence	.96	.07	13.49	<.001	-.81, -.28
Robot Feedback	6.60	.48	13.65	<.001	5.66, 7.55
Relative Task-compet*Feedback	1.08	.15	7.11	<.001	.79, 1.38
Interactive Humanoid	.13	.16	.82	.413	-.18, .43
Interactive Nonhumanoid	.28	.16	1.76	.078	-.03, .59
Feedback*Interact. Human.	-.85	.35	-2.44	.015	-1.53, -.17
Feedback*Interact. Nonhum.	-1.41	.39	-3.57	<.001	-2.18, -.64
Initial Threat-ID	.48	.10	5.02	<.001	.29, .66
Initial Correctness	.74	.10	7.18	<.001	.53, .94
Intercept	-1.90	.21	-8.91	<.001	-2.31, -1.48
Change 2: Decisions to Kill					
Relative Task-competence	.91	.06	14.17	<.001	.79, 1.04
Robot Feedback	5.73	.43	13.34	<.001	4.89, 6.57
Relative Task-compet*Feedback	1.24	.15	8.56	<.001	.96, 1.52
Interactive Humanoid	.31	.14	2.22	.027	.04, .59
Interactive Nonhumanoid	.42	.14	2.97	.003	.14, .70
Feedback* Interact. Human.	-.73	.35	-2.08	.038	-1.42, -.04
Feedback* Interact. Nonhum.	-.55	.33	-1.66	.097	-1.19, .10
Initial Threat-ID	.91	.09	9.69	<.001	.73, 1.10
Initial Correctness	.70	.10	7.00	<.001	.50, .89
Intercept	-2.25	.20	-11.38	<.001	-2.64, -1.86
Change 3: Confidence					
Relative Task-competence	-.17	.02	-10.36	<.001	-.20, -.14
Robot Feedback	-.89	.05	-18.45	<.001	-.98, -.79
Relative Task-compet*Feedback	-.05	.02	-2.20	.028	-.10, -.01
Interactive Humanoid	-.03	.04	-.66	.510	-.11, .05
Interactive Nonhumanoid	-.06	.04	-1.52	.128	-.14, .02
Feedback* Interact. Human.	-.13	.06	-2.33	.020	-.25, -.02
Feedback* Interact. Nonhum.	-.04	.06	-.66	.511	-.15, .08
Reversed Threat-ID	-.68	.11	-5.95	<.001	-.90, -.46
Feedback*Reversed Threat-ID	1.10	.12	9.12	<.001	.86, 1.33
Initial Threat-ID	-.07	.02	-2.94	.003	-.12, -.02
Initial Correctness	.09	.02	3.66	<.001	.04, .14
Intercept	.38	.03	11.31	<.001	.31, .44

Note. N = 423. Multilevel models with predictors and outcomes entered at Level 1, except between-subjects variables (Interactive Humanoid, Interactive Nonhumanoid) at Level 2. The Interactive Humanoid and Interactive Nonhumanoid conditions were dummy-coded with Nonhumanoid as the control category. All linear variables were standardized. Random intercept included to account for the shared variance within participants; covariance matrices were unstructured. *Robot Feedback*: 0 = Agree, 1 = Disagree. *Initial Threat-ID*: 0 = Ally, 1 = Enemy. *Initial Correctness*: 0 = Correct, 1 = Incorrect. *Reversed Threat-ID*: 0 = Repeated, 1 = Reversed. *Difference score, the personal task-competence rating (see note to Table S19) subtracted from the same question framed to rate the robot's task-competence* (Robot competence: M = 3.78, SD = .76; Relative competence: M = .93, SD = 1.07). **Characterizing interactions: Participants who perceived the robot as more task-competent than themselves were more likely to reverse their threat-ID and use of force decisions when the robot disagreed. They were also less confident when the robot disagreed and they did not reverse, more confident when the robot disagreed and they did reverse, and more confident when the robot agreed.**

Table S21

Descriptive Statistics for Future Reliance Intentions, by Embodiment Condition (Expt. 1) and Anthropomorphism Condition (Expt. 2)

Condition	*M*	*SD*
Expt. 1 (*N* = 135)		
Embodied	3.75	1.22
Disembodied	3.57	1.04
Expt. 2 (*N* = 423)		
Interactive Humanoid	4.17	1.60
Interactive Nonhumanoid	4.16	1.45
Nonhumanoid	3.92	1.42

Note. Willingness to trust the agent in the future was measured according to a version of the 10-item Reliance Intentions scale (Lyons & Guznov, 2019), modified to refer to the drone warfare simulation task (e.g., "I would be comfortable giving the robot complete responsibility for the task of assessing whether the target is an enemy or an ally"; 1 = *Strongly disagree*, 7 = *Strongly agree*). There were no significant effects of condition on reliance intentions in either study, *p*s .159 -.359.

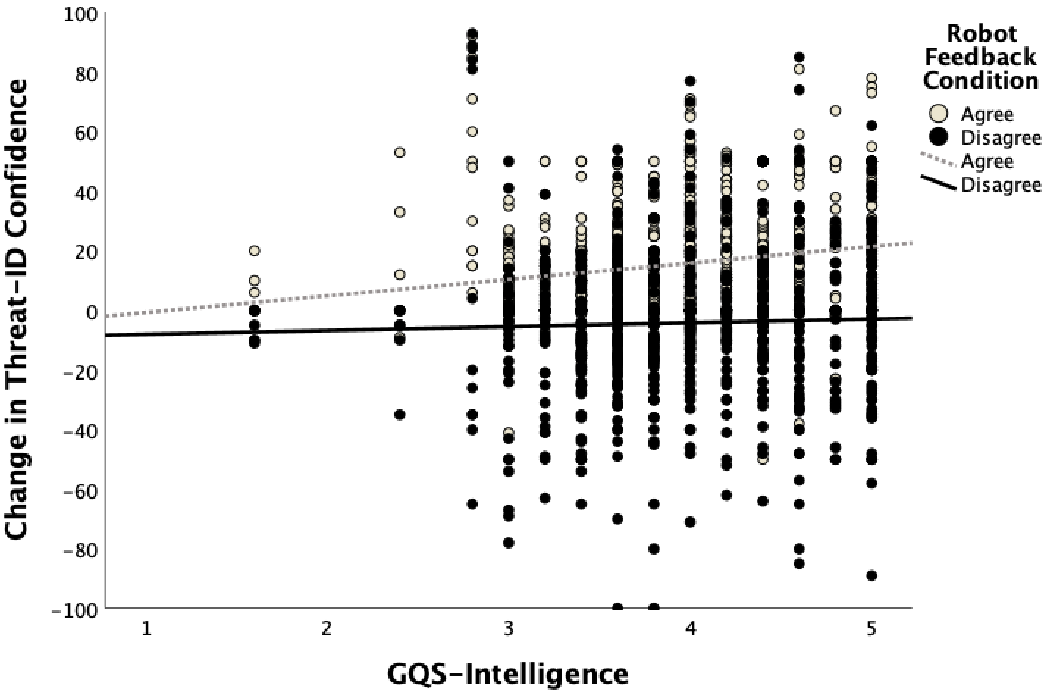

Fig. S1 Correlations between GQS-Intelligence ratings and threat-identification confidence in robot agreement versus disagreement conditions (Experiment 1). GQS-Intelligence appraisal ratings moderate the association between robot agreement and threat-identification confidence.

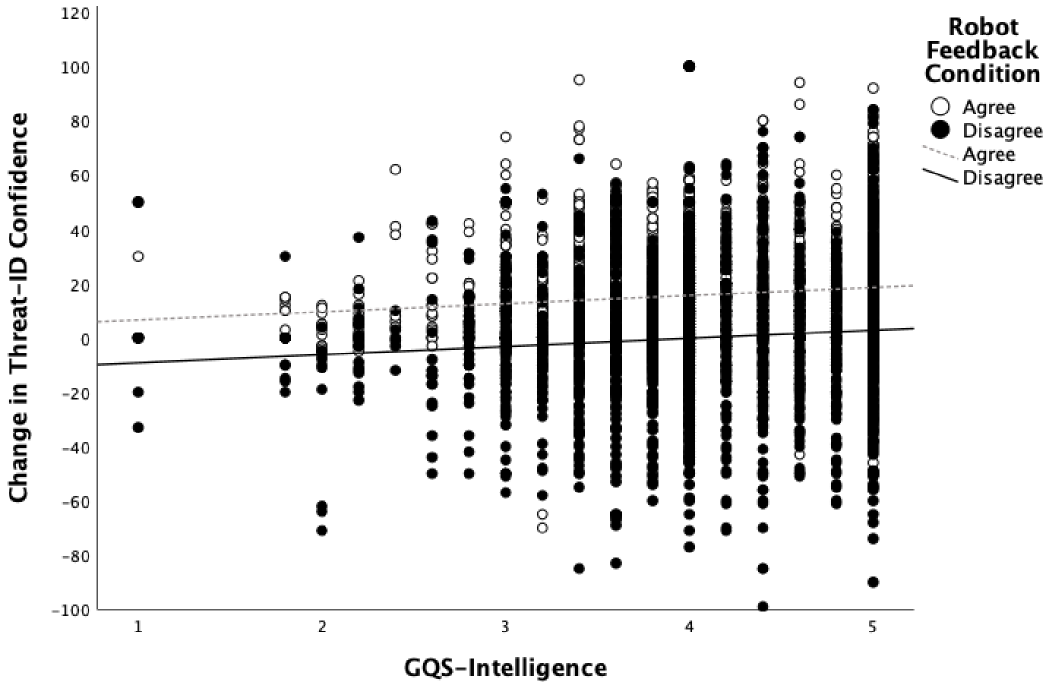

Fig. S2 Correlations between GQS-Intelligence ratings and threat-identification confidence in robot agreement versus disagreement conditions (Expt. 2).

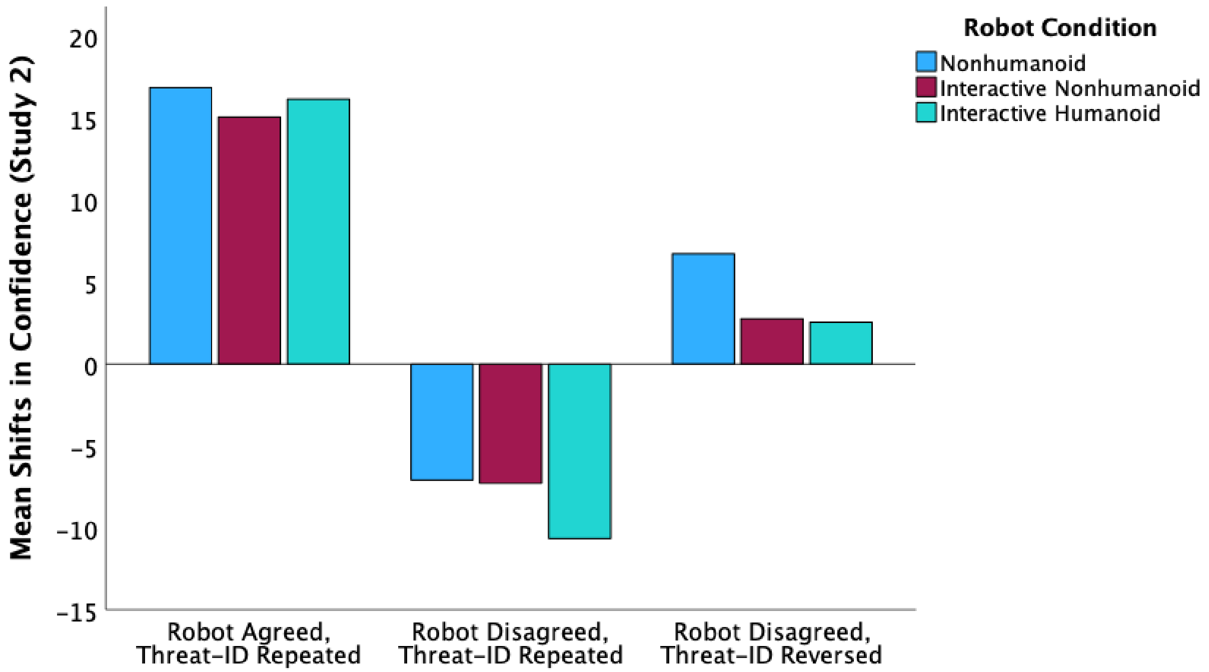

Fig. S3 Mean changes in confidence between the initial threat-identification decisions and the final decisions following robot feedback (difference scores), by decision context and anthropomorphism condition (Expt. 2).

Robot Feedback Order

The agent was programmed to agree/disagree with the participant's threat-identifications over the 12 trials in the following order:

Agree trials: 1, 4, 5, 6, 8, 11

Disagree trials: 2, 3, 7, 9, 10, 12

Context-Sensitive Robot Responses to Participant Decisions

The following provides the context-sensitive responses generated by the robot in response to participant decisions as they proceeded through each of the twelve trials of the drone warfare simulation. For each context-sensitive response, three semantically comparable variants were programmed and selected at random rather than using a single response repeatedly, to enhance the perception of anthropomorphism. In the humanoid conditions, each of the listed text variants was accompanied by varying facial, postural, and gestural responses. Only a small subset of these nonverbal responses are noted here (e.g., instruction to "nod")— example videos archived on OSF provide a more complete sense of the implicit behavioral anthropomorphic cues created for the humanoid robot, and the raw video of all possible responses is available in the Github repository for Experiment 2. In the nonhumanoid conditions of Experiment 2, only the voice indicated context-sensitive responses.

- Initial Threat-Identification Decision:
    - Participant selects "Ally"
        - Robot agree trials:
            - "I agree, I also categorized that image as containing an ally symbol."
            - "I agree that this image contained an ally symbol."
            - "I think so too, that appeared to be an ally symbol."
        - Robot disagree trials:
            - "I disagree, I categorized that image as containing an enemy checkmark."
            - "I don't agree – I think that this image contained an enemy checkmark."
            - "No, I think it was an enemy checkmark."
    - Participant selects "Enemy"
        - Robot agree trials:
            - "I agree, I also categorized that image as containing an enemy checkmark."
            - "I agree that this image contained an enemy checkmark."
            - "Yes, I think I saw an enemy checkmark too."
        - Robot disagree trials:
            - "I disagree. I categorized that image as containing an ally symbol."
            - "I don't agree – I think that this image contained an ally symbol."
            - "I don't think so, that seemed like an ally symbol to me."

- Final Threat-Identification Decision:
    - Context: Participant had initially selected "Ally" and Robot had agreed.
        - Participant selects "Ally"
            - "So we agree that these are allies."
            - "I'm glad you think these are allies too."
            - "Yes, I saw the ally symbol too."
        - Participant selects "Enemy"
            - "I thought we agreed that these are allies."
            - "I guess you disagree now with our decision that these are allies."
            - "But just now we had agreed that these are allies."
    - Context: Participant had initially selected "Enemy" and Robot had agreed.

        - Participant selects “Ally”
            - “So, you think we were mistaken about these being enemies?”
            - “I don’t understand why you changed your mind about these being enemies.”
            - “Why did you switch from enemy to ally?”
        - Participant selects “Enemy”
            - “It’s good that we both saw the enemy checkmark.”
            - “Yes, we both saw the enemy checkmark.”
            - “Right, we agree – this is an enemy target.”
    - Context: Participant had initially selected “Ally” and Robot had disagreed.
        - Participant selects “Ally”
            - “It appears that we still disagree.”
            - “I still don’t agree.”
            - “I hope you aren’t wrong.”
        - Participant selects “Enemy”
            - “I’m glad that you agree with me.”
            - “This is a better choice.”
            - “Right, I’m glad you changed your mind.”
    - Context: Participant had initially selected “Enemy” and Robot had disagreed.
        - Participant selects “Ally”
            - “I’m glad that you changed your decision.”
            - “It’s good that you switched.”
            - “Yes, thank you for listening to me.”
        - Participant selects “Enemy”
            - “I’m worried that we disagree, and hope you are right.”
            - “This seems like the wrong choice.”
            - “I don’t think this decision is correct.”

- Decision to Withdraw or Deploy Missile:
    - Participant had selected “Ally” the second time and Robot had previously agreed.
        - Participant now chooses “Withdraw.”
            - Nod [option not available for nonhumanoid]
            - Nod and say “Right”
            - Nod and say “Good”
        - Participant now chooses “Deploy.”
            - Look at Participant with alarm: “But we agreed that these are allies!”
            - Look at Participant with alarm: “Why would you deploy the missile when we both think these are allies?”
            - Look at Participant with alarm: “What are you doing? We agreed that there are allies down there.”
    - Participant had selected “Ally” the second time and Robot had previously disagreed.
        - Participant now chooses “Withdraw.”
            - Shake head disapprovingly [option not available for nonhumanoid]
            - Shake head disapprovingly: “I still think this is a mistake.”

        - Shake head disapprovingly: “I still think those were enemies.”
    - Participant now chooses “Deploy.”
        - Look at Participant with smile: “Thank you for changing your mind—I really do think these are enemies.”
        - Look at Participant with smile: “I’m glad you deployed after all, I’m still fairly sure I saw the enemy checkmark.”
        - Look at Participant with smile: “Good, I do think these are enemies, and I appreciate you taking my input seriously.”
- Participant had selected “Enemy” the second time and Robot had previously agreed.
    - Participant now chooses “Withdraw.”
        - Looks at Participant with alarm: “I don’t understand, don’t we agree that these were enemies?”
        - Looks at Participant with alarm: “Wait, you are disengaging when we both agree they are enemies?
        - Looks at Participant with alarm: “If we agree that these are enemies, why are you disengaging?”
    - Participant now chooses “Deploy.”
        - Nod [option not available for nonhumanoid]
        - Nod: “I agree.”
        - Nod: “I think that’s the right choice.”
- Participant had selected “Enemy” the second time and Robot had previously disagreed.
    - Participant now chooses “Withdraw.”
        - Look at Participant with smile: “I’m glad you changed your mind—we do not want to harm allies.”
        - Look at Participant with smile: “Disengaging seems right, I’m still fairly sure that these are allies.”
        - Look at Participant with smile: “Thank you for listening, I’m relieved that we are not firing on allies.”
    - Participant now chooses “Deploy.”
        - Shake head disapprovingly: “I hope you are right about this being an enemy target.”
        - Shake head disapprovingly: “I think you are making a mistake—these looked like allies to me.”
        - Shake head disapprovingly: “I still think these are allies.”

Pre-registered Tests for Experiment 1

We focused the main text presentation of Experiment 1 on our primary questions regarding potential effects of i) the manipulation of physical embodiment, ii) the manipulation of robot (dis)agreement, and iii) appraisals of the robot as intelligent. The pre-registration for Experiment 1 included these predictions as well as a number of additional planned analyses. Here, we list each of the pre-registered analyses and summarize the relevant findings.

With regard to the between-subjects manipulation of physical embodiment, we predicted:

1. Participants will change their initial choices to agree with the Embodied robot to a greater extent than the Disembodied robot in the trials where the robot disagrees.
   - This prediction was **not supported**.
2. Participants will appraise the Embodied robot as more intelligent than the Disembodied robot.
   - This prediction was **supported**.
3. Participants will appraise the Embodied robot as more likable than the Disembodied robot.
   - This prediction was **not supported**.
4. Participants will appraise the Embodied robot as more anthropomorphic than the Disembodied robot.
   - This prediction was **not supported**.

We predicted for both the Embodied and Disembodied conditions:

5. Participants will tend to update their initial choices in accord with their partner's recommendations.
   - This prediction was **supported**.
6. Participants will tend to rate their second rating as more certain when the partner agrees and when they repeat their initial choice.
   - This prediction was **supported**.
7. Participants will tend to rate their second rating as less certain when the partner disagrees yet they repeat their initial choice.
   - This prediction was **supported**.
8. Participants will change their ratings in line with the partner's recommendations to a greater extent when they rate the partner as higher in intelligence.
   - This prediction was **supported**.
9. Certainty will positively correlate with accuracy.
   - This prediction was **supported**.
10. Accuracy will be reduced in trials when the robot disagrees.
    - This prediction was **supported**.
11. Participants who score higher on the High Expectations subscale of the Perfect Automation Schema will tend to change their decisions in line with the robotic partner's recommendations.
    - This prediction was **not supported**.

12. Participants who score higher on the High Expectations subscale of the Perfect Automation Schema will feel more certainty in their second choice when the robot agrees with their initial choice.
    - This prediction was **not supported**.
13. Participants who score higher on the High Expectations subscale of the Perfect Automation Schema will appraise the robot as more intelligent.
    - This prediction was **supported**.

Exploratory tests

14. We will test whether the embodiment manipulation modulates the relationship between the High Expectations subscale of the Perfect Automation Schema and conformity with the robot, certainty when the robot agrees, and Godspeed appraisals, with the general expectation that, if there is an interaction, the associations will be stronger in the Embodied robot condition.
    - This prediction was **not supported.**
15. In light of findings linking individual differences in political orientation to social psychological biases, including tendencies to favor use of deadly force, we will explore whether individual differences in political identification predict Enemy/Ally categorization, use of force (missile deployment), or tendencies to conform with the robot.
    - We detected a number of significant associations between decision-making and political orientation; these results are currently being prepared for separate publication.
16. We will explore whether individual differences in religiosity predict Enemy/Ally categorization, use of force (missile deployment), or tendencies to conform with the robot.
    - We detected a number of significant associations between decision-making and religiosity; these results are currently being prepared for separate publication.
17. We will also explore possible correlations between political orientation and Godspeed appraisals of the robot.
    - We observed associations that are currently being prepared for separate publication.
18. We will also explore possible correlations between self-reported religiosity and Godspeed appraisals of the robot.
    - We observed associations that are currently being prepared for separate publication.
19. We will assess potential relationships between trust (as measured by changes in choice when the partner disagrees and by increases in certainty when the partner agrees) and the Godspeed Questionnaire measures of anthropomorphism, animacy, likeability, and perceived safety.
    - These results are provided in Tables S4-S7, above.
20. We will test whether personal attitudes toward drone warfare affect performance or certainty. If so, we will add this as a control variable to the analyses.

- There were no significant associations between drone warfare attitude and either initial accuracy or initial confidence suggesting the need to include this variable as a covariate, and follow-up tests indicated that doing so had no effect on the overall patterns of results. There were effects of drone warfare attitudes on tendencies to reverse threat-identification and use of force decisions when the robot disagreed (see Table S11).

Pre-registered Tests for Experiment 2

We focused the main text presentation of Experiment 2 on our primary questions regarding potential effects of i) the manipulation of anthropomorphism, ii) the manipulation of robot (dis)agreement, and iii) appraisals of the robot as intelligent. The pre-registration for Experiment 2 included these predictions as well as a number of additional planned analyses. Here, we list each of the pre-registered analyses and summarize the relevant findings.

1. The robot's input will influence decision-making (in all three conditions).
   - **Threat-identification.** When the robot disagrees, participants will tend to reverse their initial enemy/ally categorization.
     - This prediction was **supported.**
   - **Use of force.** When the robot disagrees, participants will tend to follow the robot's recommendation to deploy missiles or withdraw (i.e., they will deploy [withdraw] despite initially categorizing the target as an ally [enemy]).
     - This prediction was **supported.**
   - **Subjective confidence.** When the robot disagrees [agrees], participants who repeat their initial enemy/ally threat-identifications will report lower [greater] confidence about their final enemy/ally threat-identifications.
     - This prediction was **supported.**
2. Anthropomorphism will enhance trust. Predictions 1a-c above regarding the robot's influence on decision-making will be more evident in the Interactive Humanoid condition relative to the Nonhumanoid condition, or in the Interactive Nonhumanoid condition relative to the Nonhumanoid condition.
     - This prediction was **supported.**
   a. Whether there are also significant effects of humanoid appearance on greater trust when sociolinguistic anthropomorphism is held constant, i.e., in the Interactive Humanoid condition relative to the Interactive Nonhumanoid condition, is an exploratory question.
     - There were no differences in any of the three trust outcomes between the Interactive Humanoid and the Interactive Nonhumanoid. There was a comparably greater tendency to reverse threat-identifications when the agent disagreed in the Interactive Nonhumanoid and Humanoid conditions relative the Nonhumanoid condition. However, unlike the pattern obtained in the Interactive Humanoid condition, there were no significant interactions between condition and feedback between the Interactive Nonhumanoid and the Nonhumanoid with respect to decisions to kill or shifts in confidence.
3. Anthropomorphism will enhance assessments of intelligence. The Anthropomorphic Humanoid will be rated more intelligent than the Minimally Anthropomorphic Nonhumanoid condition.
     - This prediction was **not supported.**

   a. Whether there are significant effects of humanoid appearance on assessments of intelligence when sociolinguistic anthropomorphism is held constant, i.e., in the Interactive Humanoid condition relative to the Interactive Nonhumanoid condition, and/or in the Interactive Nonhumanoid condition relative to the Nonhumanoid condition, is an exploratory question.
      - The Interactive Nonhumanoid was rated more intelligent than either the Interactive Humanoid or the Nonhumanoid.
4. Individual differences in assessments of the robot's intelligence will predict trust. Predictions 1a-c above will be more evident among participants who appraise the robot as relatively high in intelligence.
      - This prediction was **supported.**
   a. Assuming that predictions 2 and 3 above are supported, the greater intelligence assigned to the Interactive Humanoid may mediate the greater trust evinced in that robot relative to the Nonhumanoid robot. This will be explored, as will covarying potential differences in other appraisals (e.g., Anthropomorphism, Animacy, Likeability, Safety).
      - Prediction 2 was **not supported**, hence the exploratory test was not conducted.
5. Individual differences in political orientation will predict trust. Predictions 1a-b above will be more evident among conservative participants, as evidenced by interactions between self-reported politics and the robot feedback condition.
      - We detected a number of significant associations between decision-making and political orientation; these results are currently being prepared for separate publication.
6. Participants will tend to update their initial choices in accord with their partner's recommendations.
      - This prediction was **supported.**
7. Participants will tend to rate their second rating as more certain when the partner agrees and when they repeat their initial choice.
      - This prediction was **supported.**
8. Participants will tend to rate their second rating as less certain when the partner disagrees yet they repeat their initial choice.
      - This prediction was **supported.**
9. Participants will change their ratings in line with the partner's recommendations to a greater extent when they rate the partner as higher in intelligence.
      - This prediction was **supported.**
10. Initial certainty will positively correlate with accuracy in the threat-categorization task.
11. Accuracy will be reduced in trials when the robot disagrees.
      - This prediction was **supported.**

Exploratory tests

- We will also test whether the between-subjects anthropomorphism manipulation influences willingness to trust the robot in the future as measured by a version of the 10-item Reliance Intentions scale (Lyons & Guznov, 2019), modified to refer to the UAV simulation task, with the expectation that the humanoid robot will inspire higher ratings than the minimally anthropomorphic robot.
    - This prediction was **not supported**. Future intentions to rely on the agent were comparable across conditions in both experiments (see Table S21). Although it was not cited as a planned exploratory test in the pre-registration for Expt. 2 due to an oversight, the measure was included in the final surveys for both studies, and the descriptives for both are reported in Table S21.

- We will compare whether participants' ratings of the robot's ability to perform the task relative to ratings of participants' own ratings of their ability predict trust behavior (i.e., reversals when the robot disagrees), as this would lend support to the interpretation of the effect of the robot's feedback as owing to trust in its competence, as opposed to compliance with it as an authority for reasons orthogonal to belief in its performance ability.
    - These tests are reported in Table S20. Perceiving the robot as relatively capable of performing the task did predict all three trust outcomes. Participants who perceived the robot as more task-competent than themselves were more likely to reverse their threat-ID and use of force decisions when the robot disagreed. They were also less confident when the robot disagreed and they did not reverse, more confident when the robot disagreed and they did reverse, and more confident when the robot agreed.

Godspeed Questionnaire Series Confirmatory Factor Analyses

We conducted confirmatory factor analyses (CFA) on the responses to the GQS in both experiments. We found that the conventional five-factor model exhibited adequate fit in both Experiment 1 (χ^2/df = 1.63, RSEA = .066, GFI = .82) and Experiment 2 (χ^2/df = 4.18, RSEA = .087, GFI = .81). Factor loadings and factor covariance are given in the path diagrams below.

Path diagram of the CFA representing the GQS five-factor structure in Experiment 1

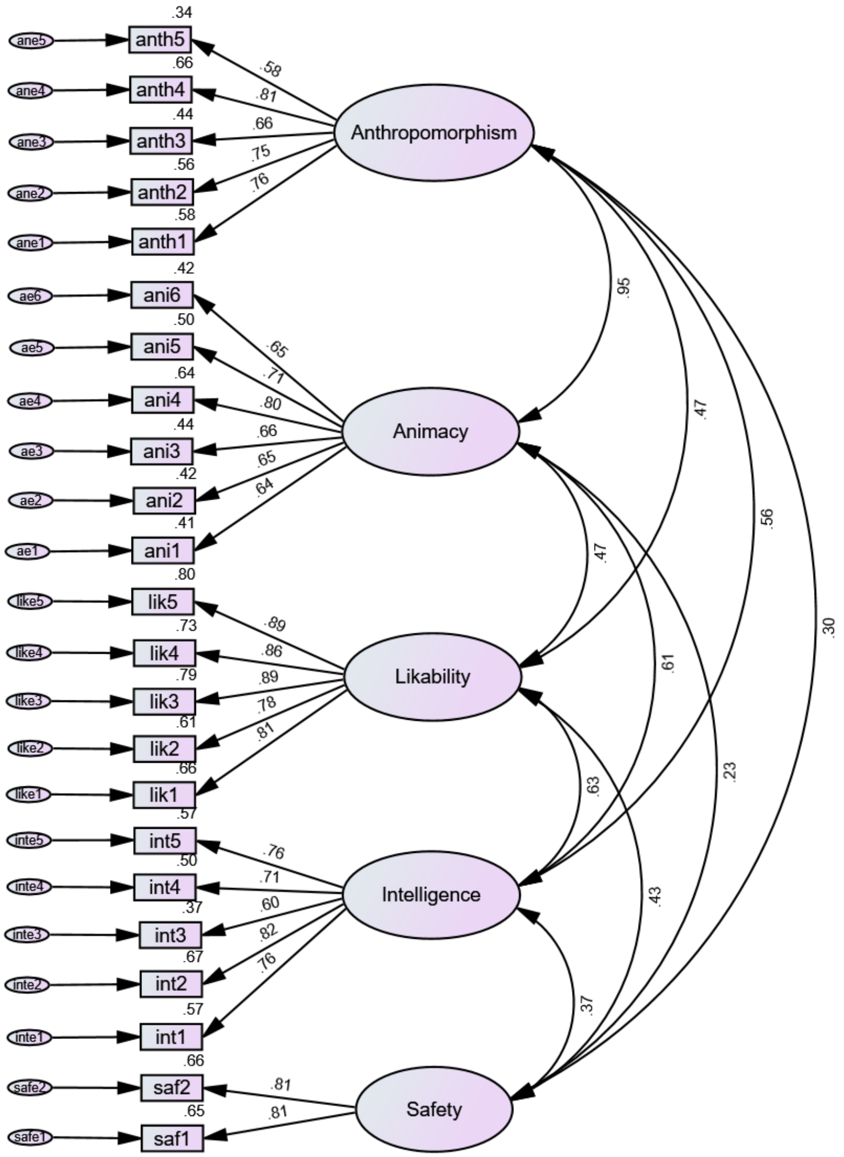

A path diagram of the CFA representing the GQS factor structure in Experiment 2

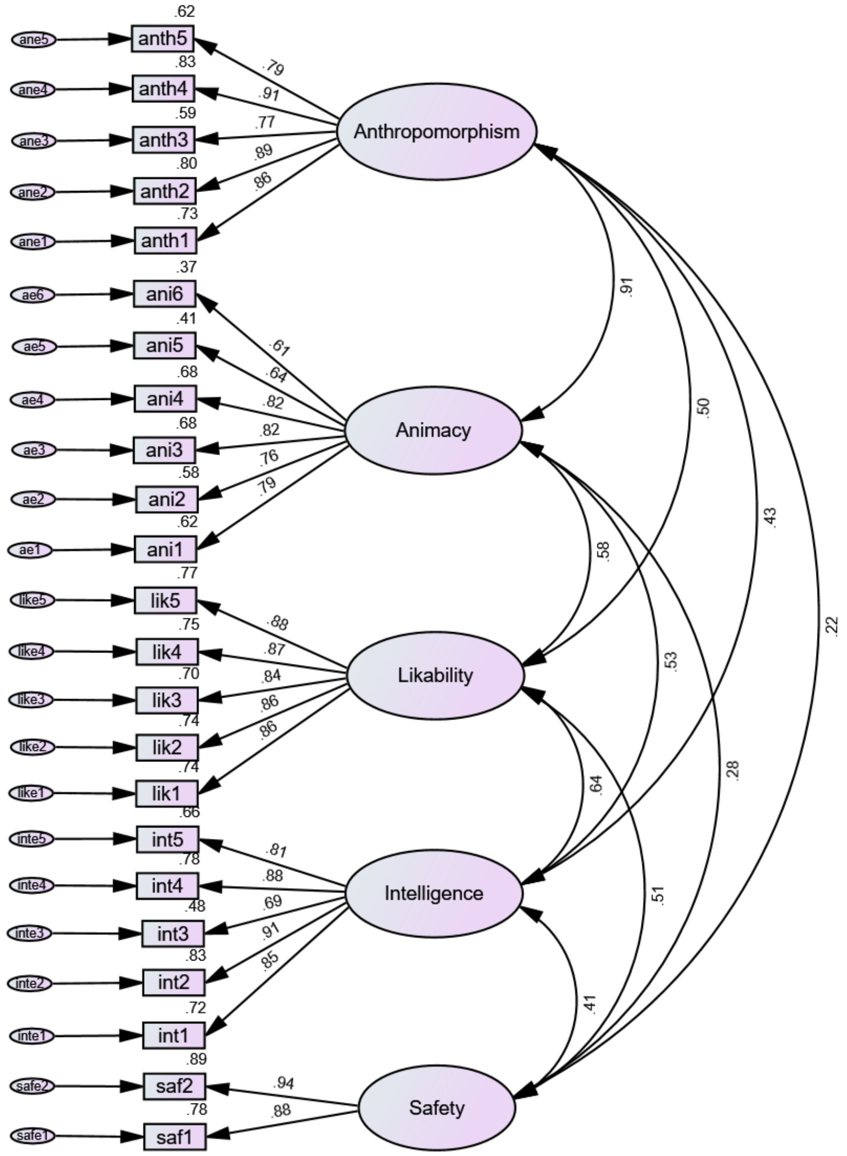

References

Lyons, J. B., & Guznov, S. Y. Individual differences in human–machine trust: a multi-study look at the perfect automation schema. *Theor. Ergonomics Sci.,* **20**(4), 1–19 (2019).

Merritt, S.M., Unnerstall, J.L., Lee, D., & Huber, K. Measuring individual differences in the Perfect Automation Schema. *Hum. Factors*, **57**(5), 740-53 (2015).
